# Cuproptosis promotes inflammatory osteolysis via GYS1-mediated glycogen metabolism

**DOI:** 10.1038/s41368-025-00408-1

**Published:** 2026-02-03

**Authors:** Lu Zhou, Hanqing Mao, Yuanhao Wen, Zhi Chen, Lu Zhang

**Affiliations:** 1 State Key Laboratory of Oral & Maxillofacial Reconstruction and Regeneration, Key Laboratory of Oral Biomedicine, Ministry of Education, Hubei Key Laboratory of Stomatology, School & Hospital of Stomatology, Wuhan University, Wuhan, China; 2 Department of Cariology and Endodontics, School and Hospital of Stomatology, Wuhan University, Wuhan, China

**Keywords:** Cell death, Mechanisms of disease

## Abstract

Copper, predominantly present in bones, plays a crucial role in bone formation. However, when copper homeostasis is disrupted, excessive copper can trigger harmful inflammation and a novel form of cell death known as cuproptosis. The impact of cuproptosis on bone metabolism remains unclear. In this study, we demonstrated that excessive copper acts as an aggravator in osteoclastogenesis and bone resorption. We observed that the expression levels of the copper importer SLC31A1 and dihydrolipoamide S-acetyltransferase (DLAT) were positively correlated with bone loss in both human chronic apical periodontitis (CAP) tissues and mouse CAP models. Untargeted metabolomics analysis and screening of glucose metabolism enzymes revealed that glycogen synthesis was inhibited during cuproptosis. Mechanistically, excessive copper hindered glycogen synthesis *via* glycogen synthase 1 (GYS1), which limited the availability of glycogenolysis-derived glucose-6-phosphate (G6P) flux into pentose phosphate pathway (PPP), and was unable to yield abundant NADPH to ensure high demand of glutathione (GSH) for macrophage survival. The inhibition of glycogen synthesis intensified cuproptosis and bone-resorption activity. Moreover, excessive copper bound to H3K27me3, which further epigenetically inhibited the gene transcription of GYS1, thereby affecting glycogen synthesis and exacerbating cuproptosis and bone resorption. Furthermore, the disruption of glycogen metabolism intensified cuproptosis and promoted inflammatory bone loss in vivo. Our finding highlighted the complex interplay among copper homeostasis, glycogen metabolism, and the osteo-immune system, suggesting new therapeutic strategies for managing inflammatory bone diseases and other copper accumulation-related conditions through the metabolic reprogramming of cells.

## Introduction

Inflammatory osteolysis represents a spectrum of clinically significant bone disorders, including rheumatoid arthritis, osteoporosis, apical periodontitis, and osteomyelitis.^1–3^ This pathological condition is characterized by progressive bone loss mediated through three interconnected mechanisms: excessive immune activation, sustained inflammatory responses, and enhanced osteoclast activity.^2,4^ The resulting excessive bone resorption disrupts normal bone architecture, leading to structural fragility and increased fracture susceptibility.^2^ Emerging evidence highlights the pivotal role of bone-immune crosstalk in bone homeostasis, where immune-derived cytokines critically regulate the functional balance between bone-resorbing osteoclasts, bone-forming osteoblasts, and mechanosensitive osteocytes.^4–6^ Of particular significance, macrophages serve dual functions in this pathophysiological cascade- acting not only as primary producers of pro-inflammatory cytokines (e.g., TNF-α, IL-1β, IL-6) but also differentiating into osteoclast precursors under specific microenvironmental cues.^7–9^ Elucidating the molecular mechanisms governing bone-immune interactions remains essential for deciphering the inflammatory bone erosion pathogenesis and identifying therapeutic targets.

Copper, a trace element predominantly localized in bone tissue, modulates collagen deposition during mesenchymal stem cell osteogenic differentiation, thereby influencing bone formation.^10–12^ Pathological conditions such as osteosarcoma, osteoarthritis, and rheumatoid arthritis alter copper homeostasis in osteoblasts, osteoclasts, chondrocytes, and synovial cells.^11^ Copper dysregulation induces detrimental inflammation and triggers cuproptosis—a unique copper-dependent cell death mechanism distinct from apoptosis, pyroptosis, necroptosis, and ferroptosis.^13^ Mechanistically, copper binds to dihydrolipoamide S-acetyltransferase (DLAT), driving disulfide bond-mediated aggregation of lipoylated DLAT and subsequent proteotoxic stress.^13–15^ Our previous study revealed that cuproptosis accelerated the progression of pulpitis.^16^ At 28 days post-pulpitis induction, significant periapical bone defects were observed, suggesting potential involvement of cuproptosis in apical periodontitis. Meanwhile, tetrathiomolybdate (TTM) mitigates cartilage degeneration in arthritis.^17^ Despite these advances, the role of cuproptosis in bone osteolysis remains poorly characterized.

Cellular copper overload not only directly induces cell death through proteotoxic stress but also exacerbates cell death processes by disrupting cellular energy and substrate metabolism.^18–21^ During cuproptosis, copper ions bind to critical tricarboxylic acid (TCA) cycle enzymes such as pyruvate dehydrogenase E1 subunit β (PDHB), inducing oligomerization-mediated inactivation that impedes TCA cycle progression, ultimately leading to metabolic collapse and cell death.^13^ Furthermore, reactive oxygen species (ROS) generated via copper catalysis oxidize metabolic enzymes, including isocitrate dehydrogenase (IDH), which further disrupts TCA cycle functionality and cellular metabolic homeostasis.^22^ In our previous study, we found overload copper inhibits pentose phosphate pathway (PPP).^23^ However, the precise mechanisms through which copper overload modulates cellular metabolism to regulate cuproptosis progression remain incompletely understood.

Glycogen metabolism, a central process governing cellular energy storage and mobilization, plays a multifaceted yet critical role in inflammatory progression.^24^ Glycogen has traditionally been regarded as an energy reservoir, where glycolysis-derived glucose-1-phosphate (G1P) initiates glycogen synthesis under the regulation of glycogen synthase 1 (GYS1) during energy surplus.^24^ Emerging studies revealed that glycogen metabolism modulates immune cell functionality, signaling through metabolic intermediates, and energy homeostasis, thereby exerting significant influence in pathologies such as cancer, diabetes, rheumatoid arthritis, and sepsis.^24–27^ Lipopolysaccharide (LPS)-stimulated macrophages enhance glycogen metabolism not only to generate nicotinamide adenine dinucleotide phosphate (NADPH), essential for inflammatory macrophage survival, but also to utilize glycogen-derived intermediates like uridine diphosphate glucose (UDPG) as signaling molecules that regulate inflammatory phenotypes.^25^ Furthermore, CD8^+^ memory T cells actively engage glycogen metabolism to support immunological memory formation and survival, contributing to antitumor immunotherapy.^28^ However, the precise mechanisms by which glycogen metabolism regulates cuproptosis progression remain to be elucidated.

In this study, we found copper overload and cuproptosis in inflammatory bone lesions. We uncovered an unexpected regulatory role of glycogen metabolism in macrophage function during cuproptosis. Excessive copper suppressed glycogen synthesis, diverting glucose-6-phosphate (G6P) away from the pentose phosphate pathway (PPP) and impairing copper detoxification. Epigenetic repression of glycogen synthase 1 (GYS1) by copper further amplifies cuproptosis and bone loss. Our finding revealed an interplay among copper homeostasis, glycogen metabolism, and bone-immune regulation, proposing novel strategies to counteract inflammatory bone resorption.

## Results

### Bone resorption in chronic apical periodontitis is associated with cuproptosis

**Fig. 1:**
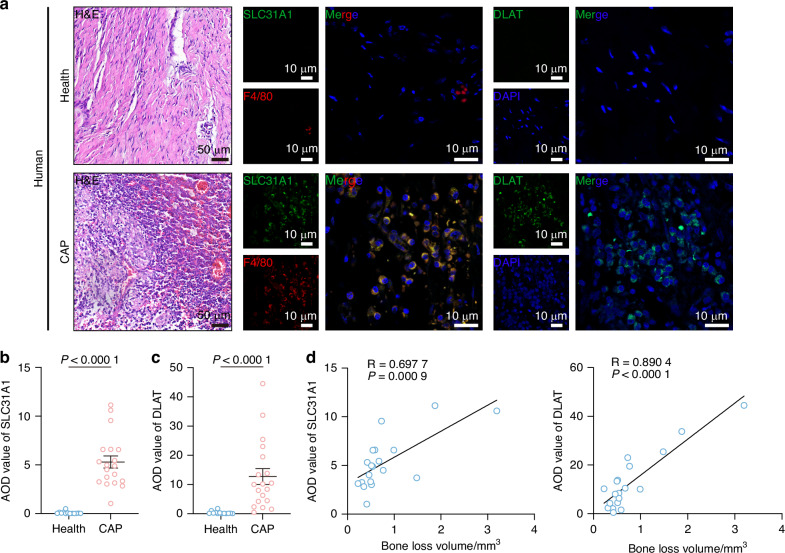
Bone resorption in chronic apical periodontitis is associated with cuproptosis. **a** Consecutive slices from 19 human chronic apical periodontitis (CAP) tissues and 13 human healthy oral mucous tissues were stained with H&E, anti-SLC31A1 antibody, anti-F4/80 antibody, anti-DLAT antibody and DAPI. **b** Difference in the average optical density (AOD) values of SLC31A1 between CAP (*n* = 19) and healthy oral mucous (*n* = 13) tissues. **c** Difference in the average optical density (AOD) values of DLAT between CAP (*n* = 19) and healthy oral mucous (*n* = 13) tissues. **d** The correlation analysis of SLC31A1 expression and apical bone loss volume (*n* = 19). The correlation analysis of DLAT expression and apical bone loss volume (*n* = 19). All error bars are mean ± SEM. *P* values were calculated by unpaired 2-tailed Student’s *t* test

Cuproptosis, a copper-dependent cell death triggered by intracellular copper overload, remains mechanistically undefined. Given copper’s skeletal enrichment,^10,11^ we investigated its role in inflammatory osteolysis. We analyzed the expression of the copper importer SLC31A1 in 19 chronic apical periodontitis (CAP) tissues and 13 healthy oral mucous tissues, revealing higher SLC31A1 levels in CAP tissues than in healthy oral mucous tissues (Fig. 1a, b). The single-cell RNA sequencing dataset revealed that macrophages exhibited the highest SLC31A1 expression levels among immune cells in periapical bone defects (Supplementary Fig. 1a, b). Immunofluorescence staining also showed that SLC31A1 was highly expressed in macrophages, which constitute the largest proportion of immune cells in CAP (Fig. 1a, b). As cuproptosis involves lipoylated DLAT aggregation,^13^ we observed elevated DLAT in CAP (Fig. 1a, c), suggesting the occurrence of cuproptosis in CAP. Positive correlations between SLC31A1/DLAT levels and bone loss (Fig. 1d) implicated copper dysregulation in inflammatory osteolysis. Inductively coupled plasma mass spectrometry (ICP-MS) confirmed elevated copper levels in CAP tissues (Supplementary Fig. 2a). To confirm copper accumulation in inflammatory osteolysis, we established mouse models for apical periodontitis (Supplementary Fig. 2b). Mouse apical periodontitis induced by pulp exposure is a well-established animal model for mimicking the pathogenesis of periapical bone erosion.^29^ Following 28-day pulp exposure, periapical lesions exhibited macrophages infiltration, SLC31A1 and DLAT upregulation (Supplementary Fig. 2c, d), confirming the occurrence of cuproptosis in inflammatory osteolysis.

### Glycogen synthesis is inhibited in cuproptotic macrophages

Elesclomol (ES), which acts as a copper ionophore, has the ability to bind copper ions and assist in their entry into cells, thereby triggering cell death that is dependent on copper.^30^ Using ES-CuCl_2_ to induce cuproptosis in bone marrow-derived macrophages (BMDMs) (Supplementary Fig. 3a), we observed dose-dependent cytotoxicity (Supplementary Fig. 3b) reversible by copper chelator TTM (Supplementary Fig. 3c). DLAT aggregation confirmed cuproptosis induction (Supplementary Fig. 3d).

**Fig. 2:**
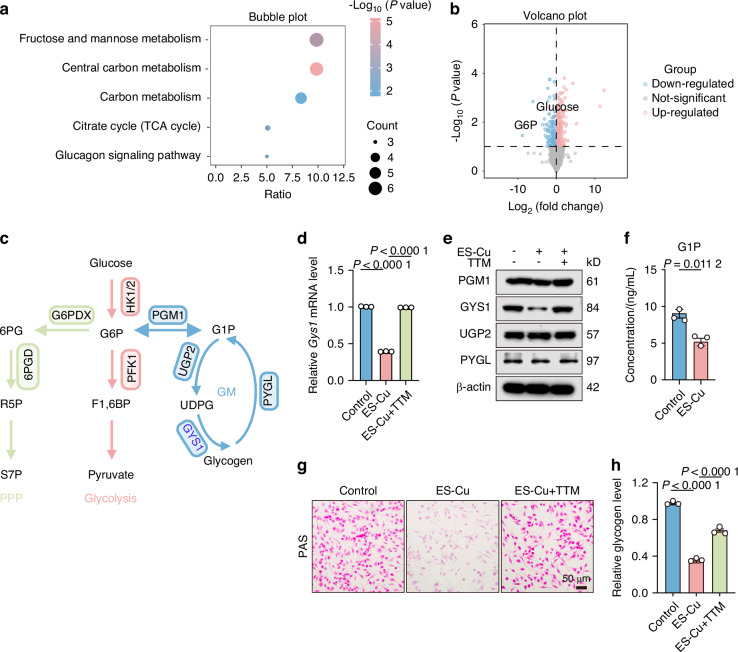
Glycogen synthesis is inhibited in cuproptosis. **a** Metabolic pathway enrichment analysis of metabolites in macrophages after ES-Cu treatment. **b** Volcano plot of differentially presented metabolites after ES-Cu treatment. **c** Diagram of glycolysis, pentose phosphate pathway (PPP), glycogen metabolism (GM). **d** *Gys1* mRNA levels in macrophages. **e** Western blot analysis of PGM1, GYS1, UGP2 and PYGL expression in ES-Cu treated macrophages. **f** G1P levels in ES-Cu-treated macrophages were detected by LC-MS. PAS staining (**g**) and colorimetric assay (**h**) were used to detect the glycogen levels in macrophages. Scale bar: 50 μm. Data are from three independent experiments. All error bars are mean ± SEM. *P* values were calculated by 1-way ANOVA followed by Tukey’s multiple-comparison test

To systematically profile the metabolic pathways during cuproptosis in macrophages, we performed untargeted metabolomics analysis on ES-Cu (150 nmol/L, 1:1) treated macrophages. Metabolic pathway analysis revealed that carbon metabolism, galactose metabolism, tricarboxylic acid cycle (TCA cycle), and glucagon signaling pathway were enriched in ES-Cu treated macrophages (Fig. 2a). Among the metabolites detected, glucose-6-phosphate (G6P), a key metabolite in glucose metabolic reprogramming was significantly downregulated in cuproptotic macrophages (Fig. 2b). We further screened enzymes involved in the glucose metabolism to obtain a comprehensive view of the transcriptomic profile associated with metabolic regulation under cuproptosis. Macrophages were treated ES-Cu at 4, 8 and 12 h respectively, and the mRNA levels of enzymes involved in glycogen metabolism during cuproptosis were examined (Fig. 2c and Supplementary Fig. 3e). Under cuproptosis, the expression of enzymes associated with glycolysis and the TCA cycle increased to meet the heightened energy demands of macrophages (Fig. 2c and Supplementary Fig. 3e). However, the enzymes involved in glycogen biosynthesis, glycogen synthase 1 (GYS1) was significantly downregulated in cuproptosis (Fig. 2c and Supplementary Fig. 3e). Consistent with this result, GYS1 was notably downregulated following ES-Cu treatment and reversed after TTM treatment (Fig. 2d). GYS1 is rate-limiting enzyme functioning in the final step of glycogen synthesis (Fig. 2c). Western blot analysis showed only GYS1 was downregulated after ES-Cu stimulation (Fig. 2e), other enzymes involved glycogen biosynthesis such as phosphoglucomutase 1 (PGM1) and UDP glucose pyrophosphorylase 2 (UGP2) and glycogen phosphorylase such as glycogen phosphorylase L (PYGL) remained unchanged after ES-Cu stimulation (Fig. 2e). We hypothesized that copper may inhibit glycogen synthesis by inhibiting GYS1. We then tested the glycogen-related metabolites in ES-Cu treated macrophages by LC-MS and found that the level of glucose-1-phosphate (G1P) was decreased compared to control, suggesting an inactive glycogen synthesis during cuproptosis (Fig. 2f). To further confirm our hypothesis about glycogen, the level of glycogen was measured and found strikingly decreased during cuproptosis and reversed by TTM, as evidenced by PAS staining and colorimetric assay (Fig. 2g, h). Together, these data suggest that glycogen synthesis is inhibited in cuproptotic macrophages *via* the inhibition of GYS1.

### Copper disrupts glycogenolysis-derived G6P flux into PPP via inhibiting GYS1

**Fig. 3:**
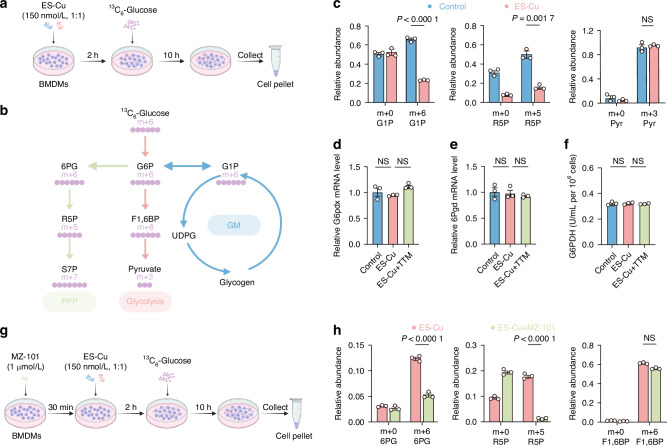
Copper disrupts glycogenolysis-derived G6P flux into PPP *via* inhibiting GYS1. **a** Schematic diagram of ^13^C_6_-glucose tracing experimental workflow in ES-Cu treated macrophages. **b** Tracer scheme illustrating the flux of ^13^C_6_-glucose to different glucose metabolic branches. Purple dots: ^13^C_6_. **c** HPIC-MS/MS was performed for m + 6-labeled G1P, m + 5-labeled R5P and m + 3-lableled pyruvate in ES-Cu treated macrophages. Relative mRNA levels of *G6pdx* (**d**) and *6Pgd* (**e**) in ES-Cu (150 nmol/L, 1:1) treated macrophages. **f** The enzymes activity of G6PDH in ES-Cu (150 nmol/L, 1:1) treated macrophages. **(g)** Schematic of workflow for ^13^C_6_-glucose tracing experiment in macrophages after MZ-101 and ES-Cu treatments. **(h)** m + 6-labeled 6PG, m + 5-labeled R5P and m + 6-labeled F1, 6BP in MZ-101 and ES-Cu treated macrophages were detected by HPIC-MS/MS. Data are from three independent experiments. All error bars are mean ± SEM. *P* values were calculated by 1-way ANOVA followed by Tukey’s multiple-comparison test

To comprehensively investigate the metabolic rerouting of glycogen under cuproptosis, we performed a ^13^C_6_-glucose stable isotope tracing experiment to assess glucose utilization in macrophages. We introduced [U_6_]-^13^C-glucose into cultured medium after ES-Cu treatment (Fig. 3a). Based on each metabolite and its isotopic ratio, the ^13^C_6_ flux ratio of the efficiency data of the isotope flow of each metabolite-labeled substrate to the substance was comprehensively calculated, revealing the substitution of ^12^C_6_ in the substance as a whole (Fig. 3b). The ^13^C_6_ tracing revealed a significant reduction in m + 6 G1P after ES-Cu treatment, confirming the impediment of glycogen synthesis (Fig. 3c). As a central metabolite, G1P can be channeled to G6P which can then be directed towards various pathways: dephosphorylated to form glucose, oxidized to pyruvate as part of glycolysis, or converted to ribose-5-phosphate (R5P) through the PPP. The ^13^C_6_ tracing showed a substantial decrease in the levels of m + 5 R5P in ES-Cu treated macrophages, suggesting a blockage of G6P channeling to PPP during cuproptosis (Fig. 3c). In contrast, the levels of m + 3 pyruvate showed exhibited no change in ES-Cu treated macrophages (Fig. 3c). To explore the reason for the inhibition of PPP, we measured two enzymes involved in regulating PPP oxidation, glucose-6-phosphate dehydrogenase X-linked (G6PDX) and 6-phosphogluconate dehydrogenase (6PGD) and found no changes in ES-Cu treated macrophages (Fig. 3d, e). Moreover, the enzyme activity of glucose-6-phosphate dehydrogenase (G6PDH), a key enzyme in the PPP that converts G6P to 6PG, showed no significant alterations after ES-Cu treatment (Fig. 3f). These results suggested that the PPP itself was not inhibited, but rather that the availability of PPP substrates reduced. We then utilized the GYS1 inhibitor MZ-101 to block glycogen synthesis (Fig. 3g). The inhibition of GYS1 could prevent G6P from being channeled to m + 6 6PG and m + 5 R5P (Fig. 3h). The level of m + 6 F1,6BP remained unchanged (Fig. 3h). These findings indicated that copper disrupted glycogen synthesis via GYS1, leading to a reduced flux of G6P into the PPP.

### Glycogen metabolism-PPP regulates macrophages phenotype in cuproptosis

**Fig. 4:**
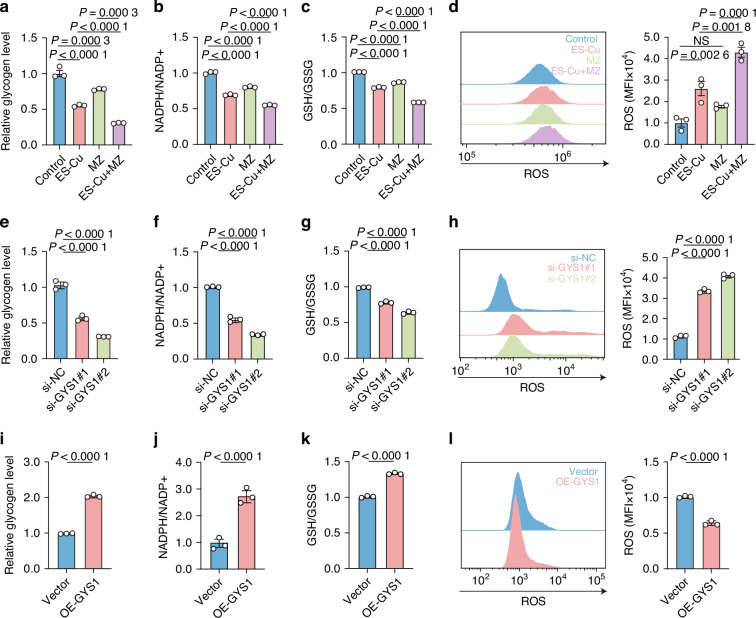
Glycogen metabolism-PPP regulates macrophages phenotype in cuproptosis. **a** Macrophages were treated with ES-Cu (150 nmol/L, 1:1) for 12 h after 1 μmol/L MZ-101 pre-treatment for 30 min. The level of glycogen was detected in MZ-101 treated macrophages. NADPH/NADP^+^ ratio (**b**), GSH/GSSG ratio (**c**) and ROS level (**d**) were analyzed. Macrophages were pretreated with GYS1 siRNA and then treated with ES-Cu (150 nmol/L, 1:1) for 12 h. The glycogen level (**e**), NADPH/NADP^+^ ratio (**f**), GSH/GSSG ratio (**g**) and ROS level (**h**) were analyzed. Macrophages were treated with ES-Cu (150 nmol/L, 1:1) after overexpressing GYS1. The glycogen level (**i**), NADPH/NADP^+^ ratio (**j**), GSH/GSSG ratio (**k**) and ROS level (**l**) were analyzed. Data are from three independent experiments. All error bars are mean ± SEM. *P* values were calculated by 1-way ANOVA followed by Tukey’s multiple-comparison test

A pivotal function of the PPP is to produce NADPH, a key molecule for converting oxidized glutathione back to its reduced form GSH, which is essential for preserving the balance of redox within the cell.^31^ Blocking glycogen metabolism by GYS1 inhibitor MZ-101 (Fig. 4a and Supplementary Fig. 4a) reduced NADPH/NADP^+^ (Fig. 4b), GSH/GSSG ratio (Fig. 4c) and increased ROS levels (Fig. 4d). In line with increased ROS levels, cell viability was strikingly diminished, indicating cell death (Supplementary Fig. 4b). Similarly, blocking glycogen metabolism by knocking down GYS1 (Fig. 4e and Supplementary Fig. 4c), NADPH/NADP^+^ ratio (Fig. 4f) and GSH/GSSG ratio (Fig. 4g) were decreased, accompanied with increased ROS levels (Fig. 4h). Cell viability was decreased (Supplementary Fig. 4d). In contrast, overexpression of GYS1 promoted glycogen production (Fig. 4i and Supplementary Fig. 4e), reversed NADPH/NADP^+^ (Fig. 4j), GSH/GSSG ratios (Fig. 4k), reduced ROS levels (Fig. 4l), and promoted cell viability (Supplementary Fig. 4f). Collectively, these findings suggest that the glycogen metabolism-PPP regulates macrophage phenotype, function in cuproptosis.

### Glycogen metabolism regulates osteoclastogenesis in cuproptosis

**Fig. 5:**
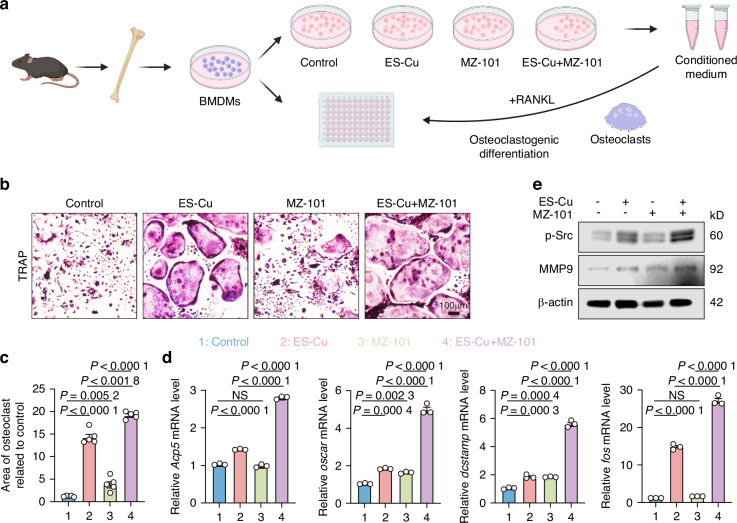
Glycogen metabolism regulates osteoclastogenesis in cuproptosis. **a** The workflow of in vitro osteoclastogenesis assay for conditioned media collection. **b** Osteoclasts were induced in conditioned media and then stained for TRAP-positive cells. Scale bar: 100 μm. **c** The quantification of TRAP-stained polynucleated (≥5 nuclei) osteoclasts that were stimulated with ES-Cu and MZ-101. **d** The mRNA levels of *Acp5*, *Oscar*, *Dcstamp* and *Fos* in BMDMs treated with ES-Cu (150 nmol/L, 1:1) after 1 μmol/L MZ-101 pretreatment. **e** Western blot analysis of p-Src and MMP9 expression in macrophages after ES-Cu and MZ-101 treatment. Data are from 3 independent experiments. All error bars are mean ± SEM. *P* values were calculated by 1-way ANOVA followed by Tukey’s multiple-comparison test

When macrophages encounter external stimulation, they have the potential to differentiate into osteoclasts. We investigated the interplay among cuproptosis, glycogen synthesis, and osteoclastogenesis. To investigate the potential that macrophages differentiate into osteoclasts, we collected conditioned medium from ES-Cu pretreated macrophages and supplemented it with macrophage colony-stimulating factor (M-CSF) and RANKL to induce osteoclast differentiation (Fig. 5a). Both ES-Cu and MZ-101 treatments significantly enhanced osteoclastogenesis using TRAP staining (Fig. 5b, c). The mRNA levels of osteoclast-related genes *Acp5, Oscar, Dcstamp*, and *Fos* were remarkably increased when cultured with the conditioned medium of the ES-Cu and MZ-101 group (Fig. 5d). Western blotting showed that osteoclast-related maker MMP9 and p-Src were increased after the treatment of ES-Cu and MZ-101(Fig. 5e). Collectively, these findings suggest that the glycogen regulates osteoclastogenesis in cuproptosis.

### Copper binding to H3K27me3 epigenetically suppresses GYS1 expression

**Fig. 6:**
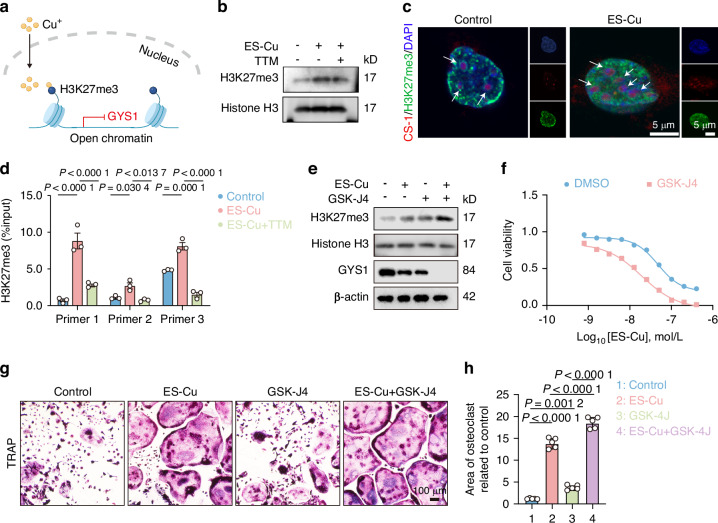
Copper binding to H3K27me3 epigenetically suppresses GYS1 expression. **a** Schematic diagram illustrating the copper binding with H3K27me3. **b** Western blot analysis of H3K27me3 expression in ES-Cu treated macrophages. **c** The co-localization of copper (red) and H3K27me3 (green) was detected by confocal. Scale bar: 5 μm. **d** ChIP-qPCR for H3K27me3 at the GYS1 promoters in macrophages. **e** Macrophages were pretreated with 1 μmol/L GSK-4J for 30 min, then treated with ES-Cu (150 nmol/L, 1:1) for 12 h. Western blot analysis of H3K27me3 and GYS1 expression in macrophages. **f** Cell viability of macrophages was analyzed. **g** The osteoclasts were induced and the representative images of TRAP staining were shown. Scale bar: 100 μm. **h** The quantification of TRAP-stained polynucleated (≥5 nuclei) osteoclasts. Data are from 3 independent experiments. All error bars are mean ± SEM. *P* values were calculated by 1-way ANOVA followed by Tukey’s multiple-comparison test

Previous studies have shown that copper (CuSO_4_) can enhance the expression of histone 3 lysine 27 trimethylation (H3K27me3), leading to the inhibition of gene transcription.^32^ We hypothesized that copper may bind to H3K27me3 to affect GYS1 transcription (Fig. 6a). We measured H3K27me3 levels after ES-Cu treatment and found a significant increase in H3K27me3 levels (Fig. 6b and Supplementary Fig. 5a). Moreover, we observed the co-localization of copper with H3K27me3 in ES-Cu treated macrophages (Fig. 6c). Chromatin immunoprecipitation (ChIP) experiments demonstrated H3K27me3 binding to the GYS1 and epigenetically suppressed GYS1 expression after ES-Cu treatment (Fig. 6d). These epigenetic reprogramming explained why copper inhibit glycogen synthesis. Additionally, we inhibited H3K27me3-demethylases with GSK-J4. GSK-J4 decreased GYS1 expression (Fig. 6e and Supplementary Fig. 5b), leading to a decline in cell viability (Fig. 6f). The GSK-J4 groups displayed substantially enhanced osteoclastogenesis compared with that of the control group (Fig. 6g, h). The mRNA levels of osteoclast-related genes were remarkably increased when cultured with the conditioned medium of the ES-Cu and GSK-J4 group (Supplementary Fig. 5c).

### The disruption of glycogen metabolism intensifies cuproptosis and inflammatory bone loss in vivo

**Fig. 7:**
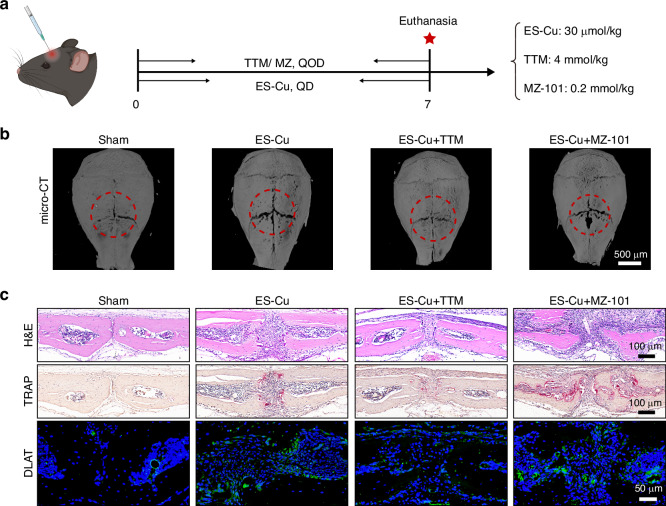
The disruption of glycogen metabolism intensifies cuproptosis and inflammatory bone loss in vivo. **a** Schematic illustration of the mouse calvarial osteolysis experimental procedure. ES-Cu (30 μmol/kg, 1:1) was administered daily to the calvaria of mice for 7 days, and mice were randomly assigned to receive TTM (4 mmol/kg) or MZ-101 (0.2 mmol/kg) via intraperitoneal injection every other day. **b** Representative micro-computed tomography (micro-CT) images of calvarial bones. Scale bars: 500 μm (*n* = 6). **c** Representative images of H&E, TRAP and anti-DLAT body staining at calvarial bones. Scale bars of H&E and TRAP: 100 μm. Scale bar of DLAT: 50 μm (*n* = 6)

To investigate the role of glycogen metabolism and cuproptosis in inflammatory bone loss in vivo, we employed calvarial osteolysis models induced by ES-Cu and mice were randomly assigned to receive TTM and MZ-101 *via* intraperitoneal injection every other day (Fig. 7a). Micro-computed tomography analysis of calvarial bones reveled that inhibiting GYS1 increased bone resorption (Fig. 7b and Supplementary Fig. 6a). TRAP staining showed a significant increase in osteoclast numbers on the calvarial bone surface in mice with GYS1 inhibition, and this increase was mitigated in mice treated with TTM (Fig. 7c and Supplementary Fig. 6b). Additionally, an increased number of macrophages on the calvarial bone surface was observed in mice with GYS1 inhibition (Supplementary Fig. 6c). Furthermore, the MZ-101 treatment groups showed significant cell death and elevated expression of DLAT, which overlapped with the macrophage region, indicating an increased occurrence of cuproptosis in macrophages (Fig. 7c and Supplementary Fig. 6c). The levels of inflammatory cytokines TNF-α and IL-6 in serum were measured by ELISA in ES-Cu calvarial osteolysis models. The results indicated that inhibition of glycogen metabolism exacerbated the upregulation of TNF-α and IL-6 in ES-Cu calvarial osteolysis models (Supplementary Fig. 6d). The increased osteoclast formation and cuproptosis were also detected in the apical periodontitis model induced by ES-Cu after MZ-101 treatment (Supplementary Fig. 7a, b). Furthermore, the levels of inflammatory cytokines TNF-α and IL-6 in serum were upregulated after MZ-101 treatment in ES-Cu induced apical periodontitis rat model (Supplementary Fig. 7c). These findings suggest that the disruption of glycogen metabolism intensifies cuproptosis and promoting inflammatory bone loss in vivo.

## Discussion

**Fig. 8:**
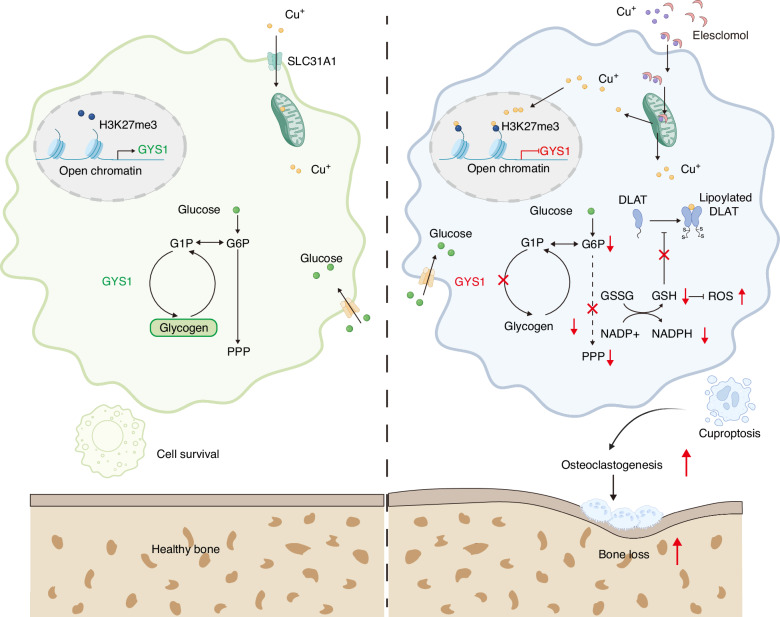
The model for regulation of cuproptosis by glycogen metabolism in macrophages. Excessive copper binds to H3K27me3, which further epigenetically inhibited the gene transcription of GYS1, thereby affecting glycogen synthesis. The inactive of glycogen synthesis limits the availability of glycogenolysis-derived G6P flux into PPP, and unable yield abundant NADPH to ensure high demand of GSH for macrophage survival

Chronic inflammatory bone diseases, including rheumatoid arthritis (RA) and osteomyelitis, are characterized by osteoclast-mediated bone erosion that correlates with clinical severity and functional impairment.^2^ Our finding identified copper overload and cuproptosis as novel pathogenic contributors to inflammatory osteolysis. Our data presented a novel perspective on excessive copper or cuproptosis as a biological aggravator of bone erosion. Through coordinated inhibition of glycogen synthase 1 (GYS1) and glycogen metabolism dysfunction, excessive copper establishes a metabolic cascade that exacerbates osteoclast activation (Fig. 8). These results revealed an intricate regulatory axis linking copper homeostasis, immune-metabolic reprogramming, and osteoclastogenesis, providing potential therapeutic targets for inflammatory bone disorders.

While copper is essential for antimicrobial defense through phagosomal bactericidal activity,^33,34^ its dysregulation induces cytotoxic effects via multiple mechanisms.^35–37^ Our data demonstrated copper accumulation (*via* SLC31A1 upregulation) and cuproptosis activation (*via* DLAT aggregation) in chronic apical periodontitis lesions, with expression levels correlating with bone loss severity. To investigate the relationship between cuproptosis and metabolic reprogramming, we performed untargeted metabolites analysis after ES-Cu stimulation. After a thorough analysis of the metabolomics data and the changes in glycolytic enzymes, we found a disruption of glycogen metabolism in macrophages after ES-Cu treatment. However, both glycolysis and the TCA cycles were significantly upregulated in response to ES-Cu induction. The shifts in glycolysis and the TCA cycles reflect both energy demands during osteoclast differentiation and compensatory responses to copper toxicity.^13^

Beyond its canonical energy storage role, glycogen functions as a guardian during cellular stress.^25,27,28,38,39^ Glycogen plays an important role in both pathological and physiological processes such as cell differentiation, redox regulation, inflammatory response, and lipogenesis.^40–43^ When cells face various stresses, glycogen can function as a reservoir, consuming itself to maintain cellular survival and homeostasis.^26,39,42^ The synthesis and breakdown of glycogen are primarily carried out by glycogen synthase (GS) and glycogen phosphorylase (GP).^44^ Macrophages regulated inflammatory responses by modulating glycogen metabolism.^25^ The gluconeogenesis-glycogenesis-glycogenolysis-PPP metabolic chain plays crucial roles in redox homeostasis.^24,25^ Our isotopic tracing data demonstrated that copper-induced GYS1 suppression disrupted the glycogenesis-PPP axis, depleting NADPH reserved essential for redox homeostasis. This impairment amplified cuproptosis by compromising glutathione regeneration capacity. The glycogen metabolism plays a significant role in the process of cuproptosis *via* GYS1 and introduced a new potential pathway to resist cuproptosis. Further research will be conducted to determine whether this also affects glycogen degradation pathways during cuproptosis in the future.

Osteoclast differentiation is initiated by macrophage-colony stimulating factor (M-CSF) and receptor activator of nuclear factor-κB ligand (RANKL).^9^ Upon binding to RANK, RANKL activates the NF-κB, MAPK, and AKT signaling pathways, as well as the key transcriptional regulators NFATc1 and c-FOS, thereby inducing gene expression essential for osteoclast formation and function. Reactive oxygen species (ROS) produced during RANKL-induced osteoclastogenesis further activate NF-κB and MAPK pathways to promote osteoclastogenesis and bone resorption.^45–47^ However, the underlying mechanism linking copper and glycogen synthase 1 (GYS1) in osteoclast differentiation remains unclear. During bacterial infection, host cells actively increase cytosolic copper levels to enhance ALPK1 kinase activity and activate NF-κB signaling pathway responsive to infection.^48^ In clear cell renal carcinoma (ccRCC), GYS1 silencing suppressed tumor growth by activating the canonical NF-κB pathway.^49^ Furthermore, glycogen metabolism regulates macrophage-mediated acute inflammatory responses, and knocking down GYS1 increased ROS release.^25^ Collectively, these findings suggest that both copper and GYS1 may regulate inflammatory pathways involving NF-κB and ROS. Further experiments are needed to elucidate these potential connections.

Copper’s interaction with histone modifications expands its regulatory repertoire beyond enzymatic cofactor roles.^50^ The copper in the cytoplasm can directly bind to ALPK1 to enhance its kinase activity.^48^ Moreover, the binding of copper to lipoylated components in the tricarboxylic acid cycle (TCA) has been shown to trigger cell death.^13^ We identify H3K27me3-mediated GYS1 repression as a novel epigenetic mechanism linking copper overload to metabolic dysfunction. This finding aligns with emerging evidence of metal-ion modulation of chromatin states,^32,51,52^ particularly through repressive marks like H3K27me3 that govern inflammatory and metabolic genes.^53,54^ The copper-H3K27me3 axis represents a potential therapeutic target to uncouple metabolic adaptation from cuproptosis progression.

Cuproptosis represents a potential therapeutic target for various diseases. Studies indicate that in tumor treatment, targeting copper ions can inhibit tumor progression by inducing oxidative stress and cuproptosis.^55,56^ In this study, we found that the cuproptosis inhibitor TTM significantly alleviates inflammatory bone loss, suggesting therapeutic potential. Therefore, elucidating the mechanism of action of cuproptosis inhibitors in inflammatory bone diseases and developing therapeutics targeting copper and cuproptosis could provide new directions and strategies for treating inflammatory bone diseases including rheumatoid arthritis, osteoporosis and apical periodontitis. Glycogen metabolism and the PPP are critical for maintaining cell survival and function. Intriguingly, our findings demonstrate that inhibiting the glycogen metabolism-PPP axis promotes inflammatory bone resorption induced by cuproptosis. These results offer a novel perspective on the role of glycogen metabolism and PPP in inflammation and cuproptosis, providing a theoretical foundation for developing therapeutic strategies targeting this pathway.

In summary, our study established glycogen metabolism as a critical regulator of cuproptosis sensitivity and osteo-immune crosstalk. The study presented a novel perspective on copper as a biological aggravator of bone erosion. Mechanically, excessive copper bound to H3K27me3, which further epigenetically inhibited the gene transcription of GYS1, thereby affecting glycogen synthesis. The inactive of glycogen synthesis limited the availability of glycogenolysis-derived G6P flux into PPP, and unable yield abundant NADPH to ensure high demand of GSH for macrophage survival. The inhibition of PPP intensified cuproptosis and promoting osteoclastogenesis. This study opens new avenues for the development of biomaterials for copper accumulation-related inflammatory diseases such as inflammatory bone diseases that target the metabolic reprogramming of cells.

## Material and methods

### Human samples

All human tissue collection protocols were approved by the Medical Ethics Committee of the School and Hospital of Stomatology, Wuhan University (Approval No. 2019LUNSHENZI [A48]). Informed consent was obtained from all participants prior to sample collection. Clinical specimens were anonymized and processed in accordance with the Declaration of Helsinki (1975). Patients diagnosed with periapical periodontitis were selected based on the following inclusion and exclusion criteria: (1) Non-smoking patients aged 21-56 without systemic diseases, (2) No antibiotic treatment for at least 1 month prior to surgery, (3) No history of previous root canal treatment, (4) Presence of obvious bone loss around the root apex detected by Cone beam CT. The diagnosis was conducted by experienced doctors based on clinical symptoms. The patients underwent periapical surgery, during which the inflamed tissue around the root apex was removed and fixed with formalin for subsequent experiments. Healthy oral mucosa tissues were obtained from residual mucosa after third molar extraction from non-smoking individuals without any signs of inflammation.

### Establishment of apical periodontitis model in vivo

7–8-week-old male mice or rats were purchased from the Model Animal Research Center of the Hubei Province (China), and were bred at SPF Animal Laboratory, Hospital of Stomatology, Wuhan University. In the ES-Cu-induced apical periodontitis model, the first left mandibular molar was perforated using a dental handpiece and 1/4 ball drill. The presence of bleeding points on the tooth surface indicated the success of the modeling. ES-Cu (40 μmol/kg, 1:1; Elesclomol, MedChemExpress, HY-12040; CuCl_2_, Aladdin, C106775) was injected into the dental pulp cavity. The access cavity was sealed with a glass ionomer. Saliva was suctioned during the operation to prevent contamination. After the procedure, water and food were avoided for 12 h. Rats additionally received PBS, MZ-101 (0.2 mmol/kg) or TTM (4 mmol/kg) through intraperitoneal injections every two days for 28 days. Besides, the dental pulp cavity was re-exposed, and ES-Cu (40 μmol/kg, 1:1) was injected 14 days after the initial exposure. Mice or rats were then euthanized on day 28 for mandibular tissue collection.

### Establishment of calvarial osteolysis model in vivo

The animal experiments were conducted in accordance with the National Institutes of Health (NIH) Guidelines for the Care and Use of Laboratory Animals. The calvarial osteolysis study protocols were approved by the Experimental Animal Ethics Committee, Stomatology Hospital of Wuhan University (S07923090E). 7–8-week-old C57BL/6 J male mice were purchased from the Model Animal Research Center of the Hubei Province (China), and were bred at SPF Animal Laboratory, Hospital of Stomatology, Wuhan University, with strict 12 h light/dark cycle. ES-Cu (30 μmol/kg, 1:1) was injected onto the calvaria of mice daily for 7 days. Mice were randomly assigned to receive TTM (4 mmol/kg) or MZ-101 (0.2 mmol/kg) by intraperitoneal injection every other day. Mice receiving only PBS were controls. Then mice were euthanized and calvarial bones were examined by micro-CT and followed by formalin fixation and decalcification in 10% EDTA for 4 weeks to prepare for histological staining.

### Cell clustering and differential expression analysis

The scRNA-seq data of five CAP samples were derived from the GSE181688 and GSE197680 datasets. Low-quality cells were filtered out from the integrated CAP samples (cells with unique feature counts over 5 000 or less than 500 and cells with over 30% mitochondrial counts). Subsequently, the data was normalized and scaled with default parameters. PCA (npcs = 50), FindNeighbors (dims = 30) and FindClusters (resolution = 0.4) were performed to identify the cell clusters. The batch effect was removed using harmony algorithm. Single cells were visualized using uniform manifold approximation and projection (UMAP). The cell types were annotated manually based on canonical markers. Visualization of feature expression was performed by DotPlot.

### Cell preparation and osteoclast differentiation

Bone marrow-derived macrophages (BMDMs) were extracted from the femur and tibias of 4- to 5-week-old C57BL/6J male mice. The cells were plated at a density of 3 × 10^6^ per mL in adhesion medium, which was composed of ES-Cu (150 nmol/L, 1:1) or indicated drug doses, along with 10% fetal bovine serum (FBS, Gibco). The cells were then incubated at 37°C in a humidified 5% CO_2_ environment for 1 day. Following this, the supernatant was collected, and non-adherent cells were removed. The collected supernatant was added to macrophages and supplemented with osteoclast-medium, consisting of 30 ng/mL of macrophage colony-stimulating factor (M-CSF, PeproTech, 315-02) and 50 ng/mL of receptor activator of nuclear factor κB ligand (RANKL, R&D Systems, 462-TEC-010). The cells were cultured at 37 °C for 7-10 days until maturation. The assay was concluded on the same day for each pair of cells. The medium was exchanged on day 3, day 5, and day 7 of cell culture. Osteoclast differentiation was assessed using TRAP staining with the acid phosphatase leukocyte kit (Sigma-Aldrich, 387 A). Images were captured using a microscope (Olympus, Japan), and the quantification of osteoclast numbers was conducted with ImageJ.

### Cell death assay

Cell death was assessed by PI staining (Beyotime, C2015M). BMDMs were seeded at densities of 4 × 10^4^ cells per well in 48-well plates. Following seeding, the cells were treated as required. For PI staining, the cells were exposed to PI working fluid for 30 min in an incubator of 5% CO_2_ at 37 °C. The PI-positive cells were visualized using a fluorescence microscope (Olympus, Japan) and quantified by Image J 1.53.

### Cell viability assay

Cell viability was analyzed with a Cell Counting Kit-8 (Biosharp, BS350C). 1 × 10^4^ BMDMs were planted in 96-well plates and allowed to adhere. Indicated concentrations of compounds were introduced to the medium for a 24 h stimulation. Post-treatment, 100 μL of fresh medium, along with 10 μL of CCK-8 solution was added to the cells and incubated for 2 h in an incubator of 5% CO_2_ at 37 °C. Absorbance at 450 nm was determined using a microplate reader (Biotek, Synergy H1).

### ^13^C_6_ tracing experiment

BMDMs pretreated with MZ-101 (1 μmol/L, 30 min) and ES-Cu (150 nmol/L, 2 h) were incubated with [U_6_]-^13^C-glucose (Sigma, 310808) for 10 h. Metabolites were extracted using methanol/water (4:1 v/v), analyzed via HPIC-MS/MS (Dionex ICS-6000 with AS11-HC column), and processed using Skyline/PROGENI/QI software.

### Histological analysis

Calvarial bones of mice were fixed overnight in 4% PFA at 4 °C followed by decalcification in 10% EDTA for 1 month until bones were pliable. The EDTA decalcification solution was refreshed every other day. The decalcified tissues were then processed dehydration in a Leica Peloris automated processor. Tissues were paraffin-embedded and cut into 5 μm slices. Sections were deparaffinized and rehydrated. Osteoclasts in mice calvarial bones were identified by staining for TRAP using an Acid Phosphatase Leukocyte Kit (Sigma-Aldrich, 387A). The abnormal areas in the calvarial bones, as indicated by H&E staining, were quantified using Image J 1.53.

### Untargeted metabolomics analysis

Metabolite extraction was performed by mixing 100 μL sample with 400 μL ice-cold extraction buffer (methanol:acetonitrile, 1:1 v/v) containing deuterated internal standards. After vortexing (30 s) and sonication (10 min, 4 °C), samples were incubated at −40 °C (1 h) for protein precipitation. Following centrifugation (12 000 r/min, RCF = 13 800 × *g*, 15 min, 4 °C), supernatants were transferred for LC-MS/MS analysis. For cellular metabolites (10⁷ cells), 1 mL extraction buffer (methanol: acetonitrile:water, 2:2:1 v/v) was added. After three freeze-thaw cycles (liquid nitrogen/RT), samples were processed as above. LC-MS/MS analysis was conducted using a UHPLC system (Vanquish, Thermo) with ACQUITY BEH Amide column (2.1 × 50 mm, 1.7 μm) coupled to an Orbitrap Exploris 120 MS. Mobile phases: (A) 25 mM NH₄Ac/NH₄OH (pH 9.75); (B) acetonitrile. MS parameters: ESI ± 3.8/−3.4 kV, sheath gas 50 Arb, aux gas 15 Arb, capillary temp 320°C. Data were processed via XCMS (R package) and BiotreeDB (v3.0).

### ChIP-qPCR

Chromatin immunoprecipitation (ChIP) Assay Kit (Active Motif, 53009) was employed to investigate the interaction between H3K27me3 and the GYS1 promoter. Cells were fixed with 1% formaldehyde on ice to crosslink the proteins bound to the chromatin DNA. Subsequently, the chromatin DNA was sheared by enzymatic force to produce DNA fragments of around 200–1 000 bp. Equal amounts of sheared DNA were employed for immunoprecipitation with either the H3K27me3 antibody (Abcam, ab6002) or an equal quantity of Rabbit IgG (Abcam, ab172730). The immunoprecipitated material was incubated with protein G Magnetic Beads, and the antibody-protein G Magnetic Beads complex was collected for subsequent reverse cross-linking. The equivalent quantity of sheared DNA without antibody precipitation was subjected to reverse cross-linking and served as input control. The DNA obtained from reverse cross-linking was used for PCR amplification. PCR was carried out using primers targeting the GYS1 promoter flanking the GYS1 binding site at 59 °C for 36 cycles. The GYS1 promoter primer sequences were listed in Supplemental Table.

### Micro-CT analysis

The mice calvarial bone tissue scanning condition was 55 kV, 200 μA with 9 μm image pixel size. The region of interest (ROI) was defined as a 6 × 6 mm^2^ calvarial area centered at the junction of the coronal and sagittal sutures (approximately 713 slices). The 6 × 6 mm^2^ regions were used for quantifying bone histomorphometric parameters including: bone surface/bone volume (BS/BV), bone volume/tissue volume (BV/TV), bone surface/ tissue volume (BS/TV) and trabecular number (Tb.N) by CTAnalyser (Bruker).

### Immunofluorescence staining

For tissues, the sections were routinely deparaffinized and rehydrated. After incubation with 3% H_2_O_2_, antigen retrieval, and blocking, sections were incubated with primary antibody overnight at 4 °C. After washing with PBS, the slides were incubated with Alexa Fluor 488 conjugated secondary antibody (Abbkine, A23220) or Alexa Fluor cy3 conjugated secondary antibody (Abbkine, A22210) for 1 h at room temperature, followed by mounted with fluormount with DAPI. Fluorescence images were acquired by a fluorescence microscope (Olympus, Japan). Relative fluorescence intensity was quantified by ImageJ 1.53. The antibodies as follows: SLC31A1 (Abmart, T510261), CD68 (Cell Signaling Technology, 97778), DLAT (Proteintech, 68303-1-lg).

For cells, treated cells were 4% paraformaldehyde-fixed for 10 min, rinsed with PBS, permeabilized with 0.2% Triton X-100 in PBS for 10 min, and blocked with serum at room temperature for 1 h. Subsequently, the cells were incubated overnight at 4 °C with primarily antibodies, followed by PBS rinsing and incubation with secondary antibodies (Abbkine, A23220) for another 1 h at room temperature. Finally, after PBS washed 3 times, the cells were stained with DAPI. All images were collected with a confocal microscope (Olympus, Japan) or fluorescence microscope (Olympus, Japan). Relative fluorescence intensity was quantitated by Image J 1.53. The antibodies as follows: H3K27me3 (Abcam, ab6002), DLAT (Proteintech, 68303-1-lg).

### Immunohistochemical staining

Sections were deparaffinized and rehydrated, and then incubated in 3% H_2_O_2_ for 10 min, boiled with citrate buffer (pH 6.0) for antigen retrieval, and then blocked with 5% serum followed by incubating overnight at 4 °C with primary antibody, including anti-F4/80 (Proteintech, 28463). Following washing, secondary antibodies and streptavidin-biotin complex (SABC) were applied with SABC-POD kits (BOSTER, SA1022). And the immune detection was performed using DAB (Dako, K3468) according to the manufacturer’s instructions. After counterstaining with hematoxylin for 1 min, the slides were mounted with neutral gum. The Aperio ScanScope CS scanner (Leica) and SlideViewer software (3D HISTECH) were utilized for panoramic scanning of immunohistochemistry.

### TUNEL staining

The sections were routinely deparaffinized and rehydrated, and then stained by a TUNEL cell apoptosis detection kit (Beyotime, C1089) according to the manufacturer’s instructions. Fluorescent images were captured using a fluorescence microscope (Olympus, Japan).

### Protein extraction and western blot

Cells were harvested and lysed with RIPA buffer containing protease inhibitor cocktail (MedChemExpress, HY-K0010). The supernatants were collected by centrifugation at 13 000×g for 15 min at 4 °C. 25 µg of protein was loaded into a 10% PAGE gel, and then subjected to electrophoresis. The separated proteins were transferred to PVDF membranes (Roche, 66898400). The membranes were then blocked with 5% non-fat milk/TBST (Biosharp) for 1 h at room temperature, followed by overnight incubation with primary antibodies at 4 °C. After three washes with TBST, the membranes were subsequently incubated with the appropriate secondary antibody diluted in TBST for 1 h at room temperature. Primary antibodies as follows: anti-PGM1 (Abcam, ab192876), anti-UGP2 (Abcam, ab154817), anti-GYS1 (Cell Signaling Technology, 3886), anti-H3K27me3 (Abcam, ab6002), Histone H3 (Proteintech, 17168-1-AP,), anti-PYGL (Proteintech, 15851-1-AP).

### Glycogen detection

Cells (1 × 10^6^ per well) were seeded in 6-well plates and treated with drugs for 24 h. The level of glycogen was measured by Glycogen Assay Kit (Abcam, ab65620) according to the manufacturer’s instructions.

### Periodic Acid-Schiff staining (PAS)

PAS staining was utilized to detect glycogen in tissues and cells by a PAS Assay Kit (Servicebio, G1008). Paraffin sections were deparaffinized with water. The sections were then immersed in periodic acid for 10–15 min, followed by rinsing in distilled water three times, each time for about 10 s. Subsequently, the sections were incubated in PAS solution Schiff reagents and protected from light for 25–30 min, and then rinsed in running water for 5 min. Counterstaining was done with hematoxylin for 1 min. Following this, the sections underwent dehydration in three changes of anhydrous ethanol for 5 min each, were cleared in xylene for 5 min, underwent an additional clearing in fresh xylene for 5 min. After neutral gum mount, the Aperio ScanScope CS scanner and SlideViewer software were used for panoramic scanning.

For cell glycogen detection, 5 × 10^5^ macrophages were cultured on cell crawling piece in 12-well plates and treated with drugs for 24 h. After fixed in 4% paraformaldehyde for 30 min, cells were washed with PBS and then stained according to manufacturer’s instructions. The cell crawling pieces were captured by an optical microscope (Leica).

### RNA extraction and real-time PCR

Cellular RNA was isolated using the RNA miniprep kit (Axygen, AP-MN-MS-RNA-250). The purity and concentration of the RNA were assessed using the Nanodrop 2000 (Thermo Scientific). Subsequently, cDNA was synthesized by reverse transcription using HiScript II Reverse Transcriptase (Vazyme, R223-01). The resulting cDNA was then diluted to a concentration of 100 ng/μL in nuclease-free water. Gene expression analysis was conducted using the QuantStudioTM 6 Flex Real-Time PCR system (Life Technologies) with ChamQ SYBR qPCR Master Mix (Vazyme, Q311). Quantitative real-time PCR was carried out in triplicates, and the data were normalized and analyzed using the ΔΔCT method. β-actin was employed as the internal reference gene. Primer sequences are available in the Supplemental Table.

### ELISA

Mouse serum and rat serum were collected and centrifuged at 4 000 r/min for 30 min. The serum levels of IL-6, TNF-α were measured with ELISA kits (Mouse, Thermo Fisher Scientific, KMC0061& BMS607-3; Rat, Thermo Fisher Scientific, ERA31RBX5& ERA56RBX5) according to the manufacturer’s instructions.

### NADPH/NADP^+^ assay and GSH/GSSG assay

The NADPH/NADP^+^ ratio was assessed utilizing the NADP/NADPH Quantification Colorimetric Kit (Abcam, ab65349), while the GSH/GSSG ratio was determined using the GSH/GSSG Quantification Colorimetric Kit (Abcam, ab138881). All measurements were conducted in accordance with the manufacturer’s guidelines.

### ROS detection

Cells (1 × 10^6^ per well) were seeded in 6-well plates and treated with MZ-101and ES-Cu. Collect the cell pellets by centrifugation. Resuspend the pellets in DMEM and centrifuge again. Discard the supernatant and retain the cell pellet. Reactive oxygen species (ROS) levels were assessed according to the manufacturer’s guidelines (Beyotime, S0033S). Prepare the ROS detection working solution in DMEM according to the kit manufacturer’s instructions. Resuspend the cells in the prepared ROS working solution and incubate in the dark at 37 °C for 20 min. Discard the ROS working solution and wash the cells twice with PBS. The fluorescence intensity was measured using a flow cytometer (Beckman) and analyzed by FlowJo.

### Plasmid constructs and transfection

GFP-tagged mouse GYS1 was cloned into the pcDNA3.1 vector. Cells were transfected with GYS1 plasmid using Lipofectamine 3000 (Thermo Fisher Scientific, L3000-015) according to the manufacturer’s instructions. All constructs were confirmed by DNA sequencing. More than 80% fluoresce-positive cells per well could be used for the following analysis.

### siRNA interference

Macrophages were transfected with 20 nmol/L siRNA (Genepharma) and Lipofectamine 3000 (Thermo Fisher Scientific, L3000-015) according to the manufacturer’s protocol. Nonspecific siRNA was used as a negative control. The following siRNA sequences were used: si-GYS1#1 sense 5’-CCCACUUCCACGRAUGGUUTT-3’, antisense 5’-AACCAUUCGUGGAAGUGGGTT-3’; si-GYS1#2 sense 5’-GCACCUGGACUUCAACCUATT-3’, antisense 5’-UAGGUUG AAGUCCAGGUGCTT-3’.

### Statistics

All experiments were conducted a minimum of 3 times. The results are presented as mean ± SEM as appropriate and were analyzed using either a 2-tailed Student’s *t* test or 1-way ANOVA, followed by Tukey’s multiple-comparison test. *P* value less than 0.05 was considered statistically significant. The analysis was performed utilizing GraphPad Prism 8.0.

The detailed description is provided in the supplementary information.

## Supplementary information

Original western blot data

Supplementary information

## Data Availability

All the data and methods are presented in the manuscript or in the Supplemental Materials. Additional data will be made available on request.
