# Supplementary figures and images for: Cuproptosis promotes inflammatory osteolysis via GYS1-mediated glycogen metabolism
Source: Int J Oral Sci. 2026 Feb 3;18:13. doi: 10.1038/s41368-025-00408-1 (PMC12864900; doi:10.1038/s41368-025-00408-1)

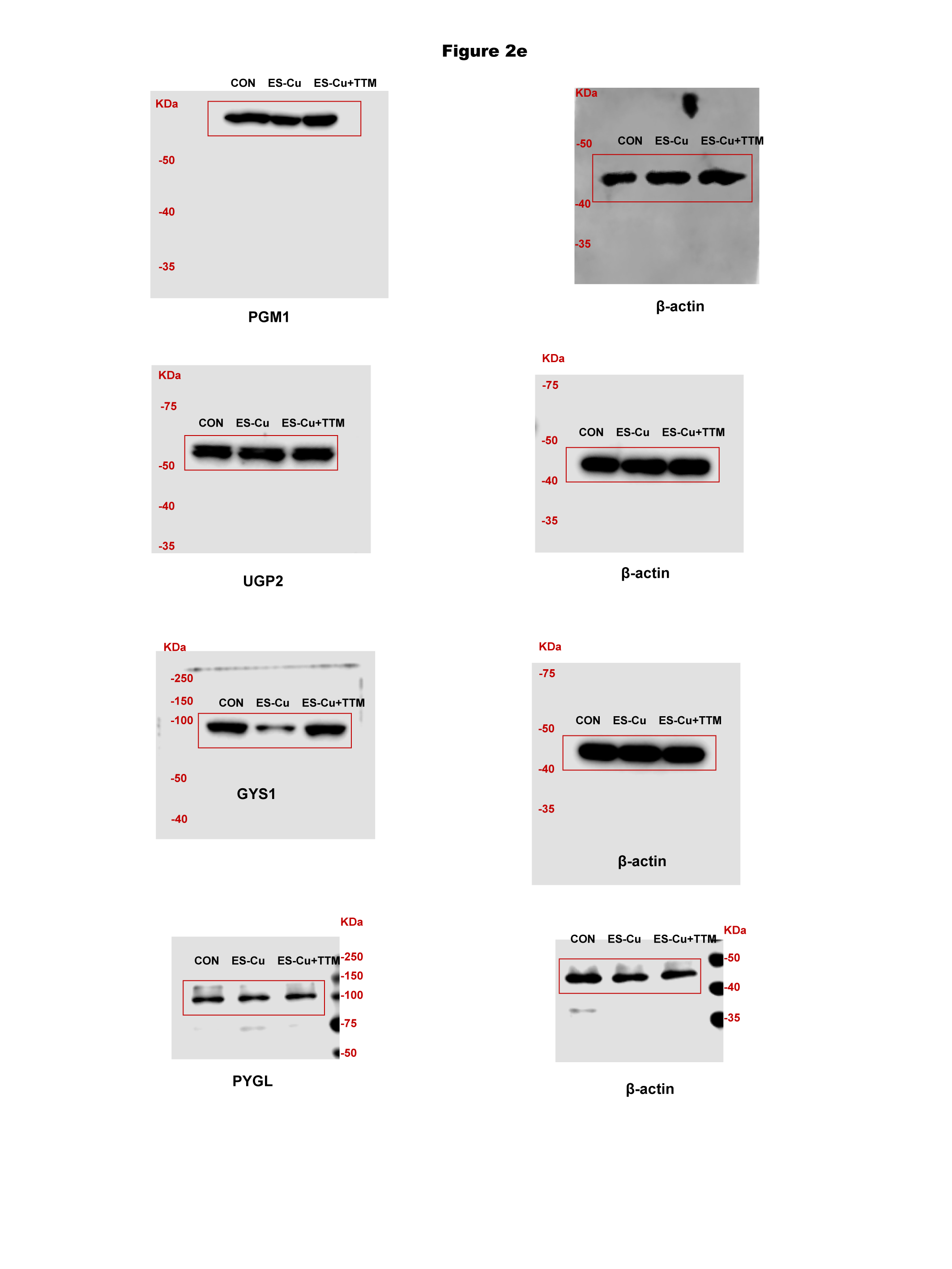


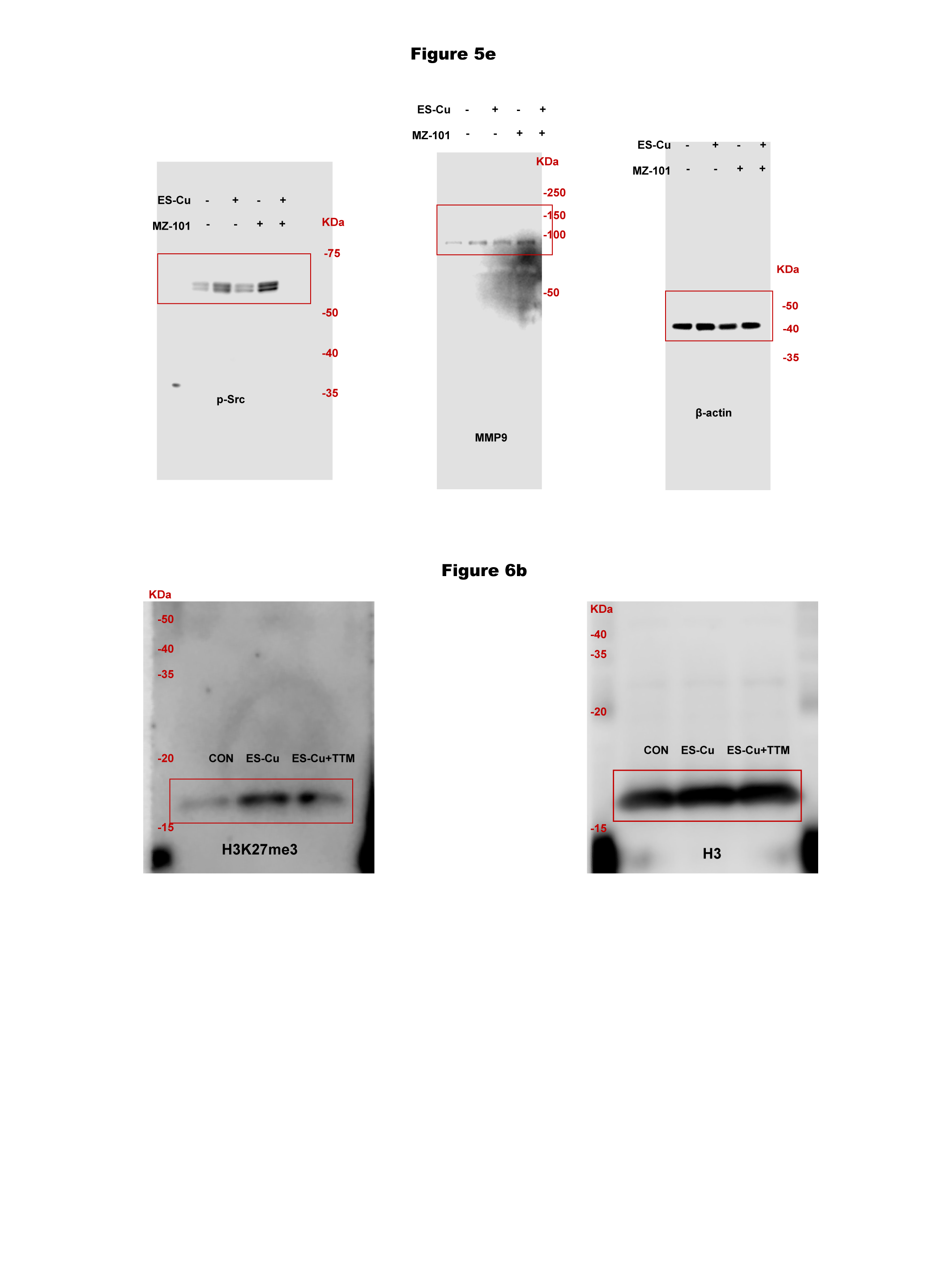

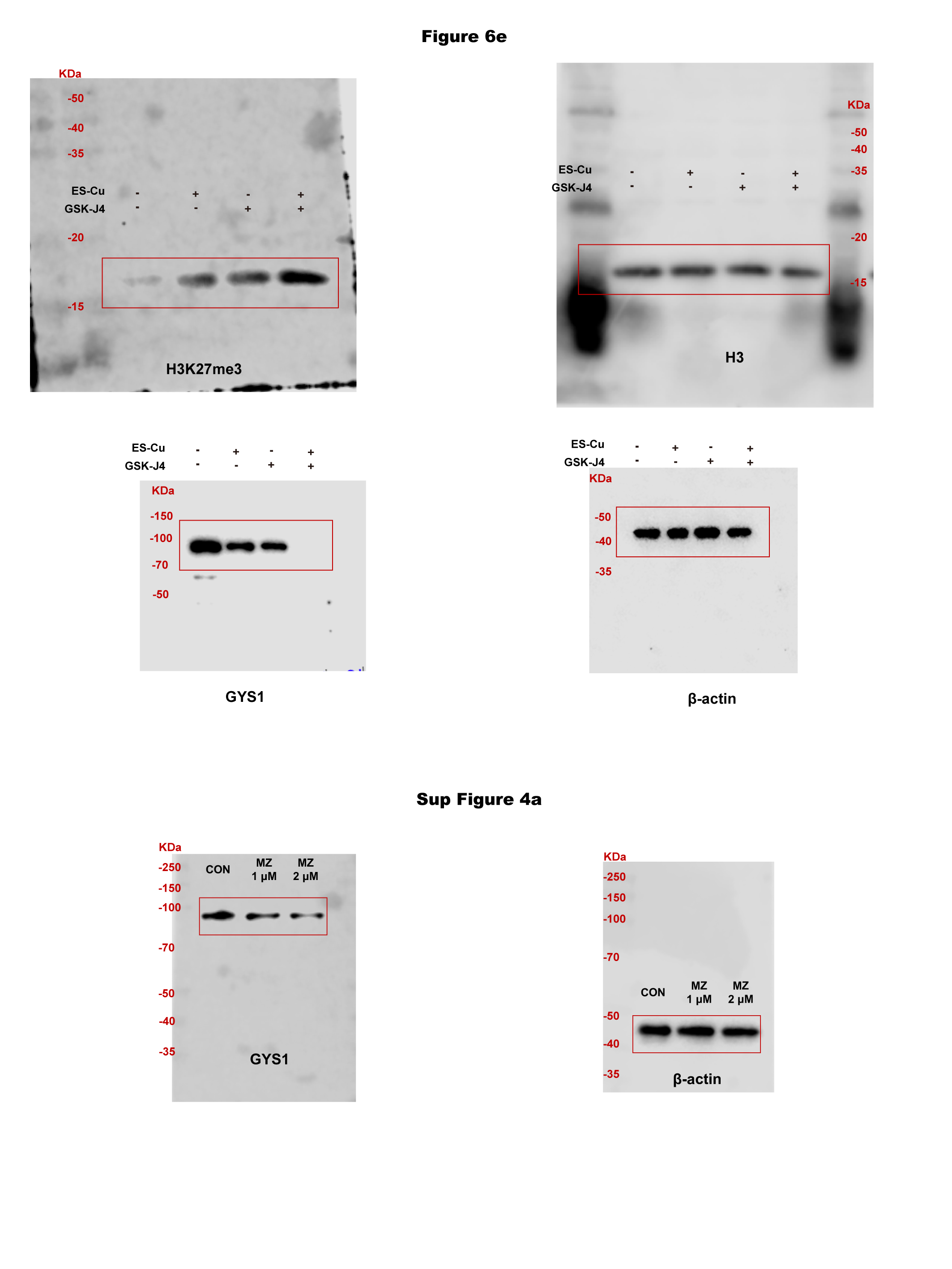

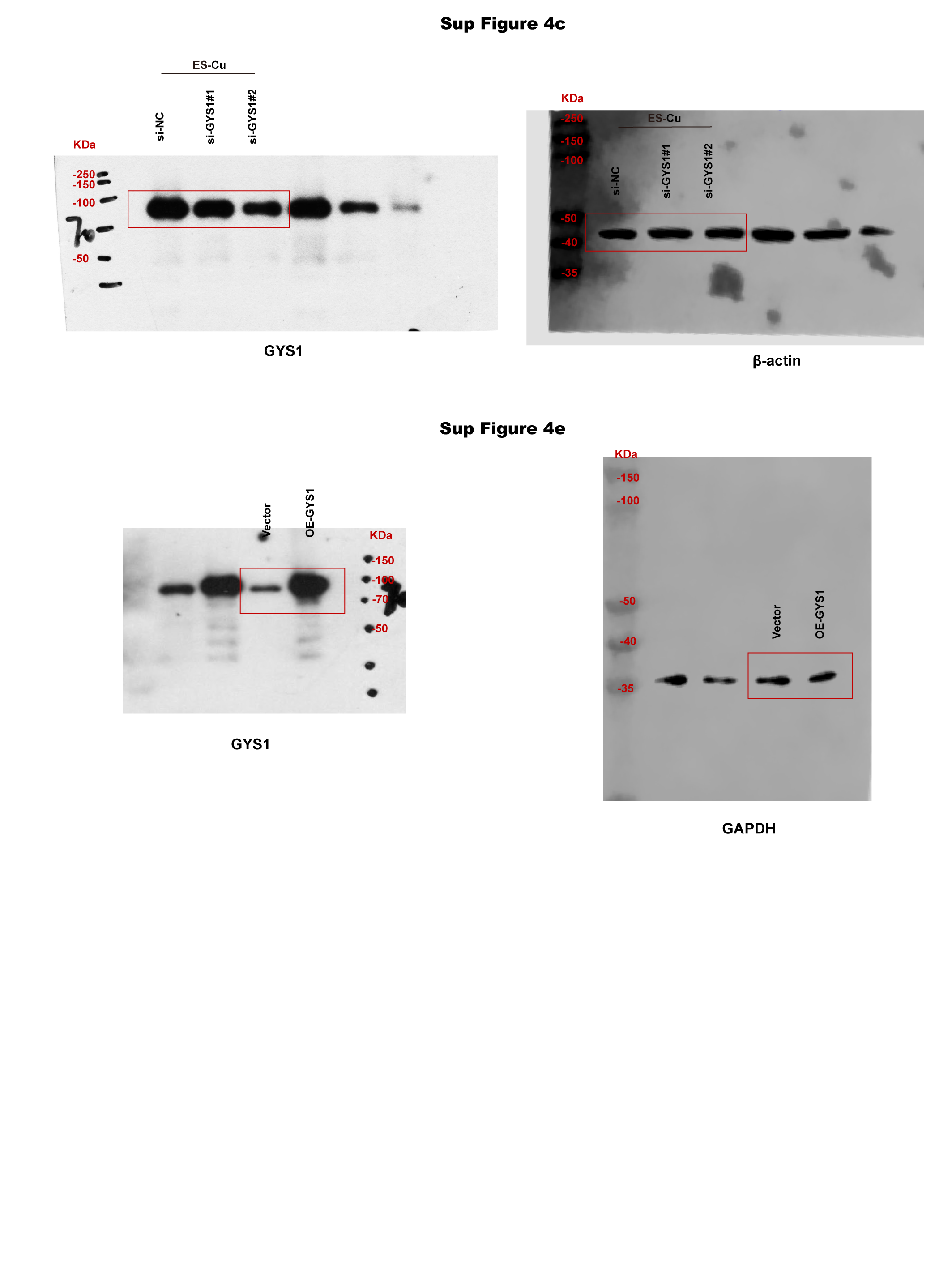

Supplement: Supplementary file 1 — Original western blot data [file 41368_2025_408_MOESM1_ESM.docx]
