# Supplementary material for: Cuproptosis promotes inflammatory osteolysis via GYS1-mediated glycogen metabolism
Source: Int J Oral Sci. 2026 Feb 3;18:13. doi: 10.1038/s41368-025-00408-1 (PMC12864900; doi:10.1038/s41368-025-00408-1)
Supplement: Supplementary file 2 — Supplementary information [file 41368_2025_408_MOESM2_ESM.docx]

**Supplementary information**

**Supplementary Figures**

**
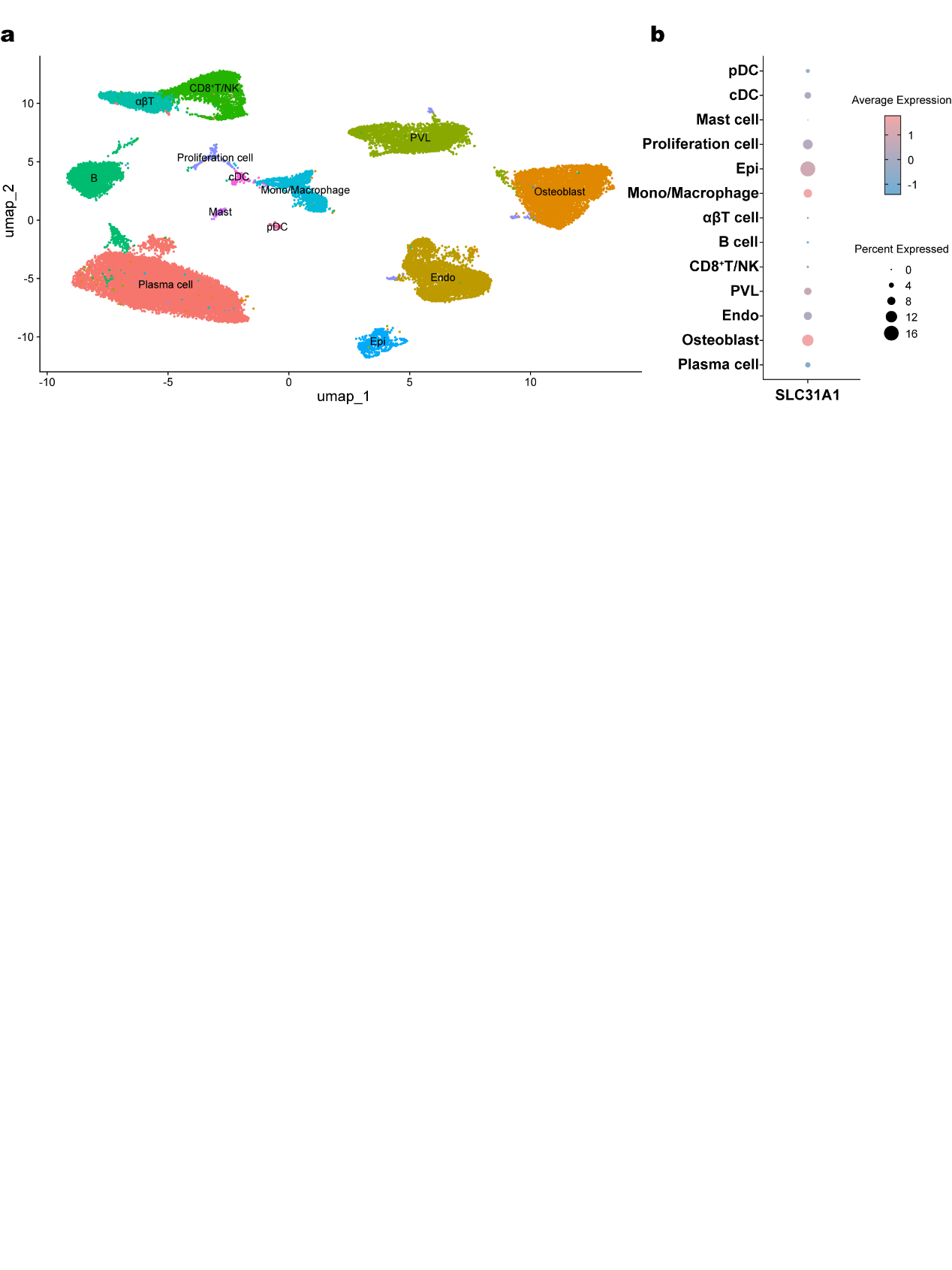
**

**Supplementary Fig 1 (a)** UMAP plot of five CAP samples. **(b)** Dot plot showing differential expression of SLC31A1 in different cell types. cDC, conventional dendritic cell; Endo, endothelial cell; Epi, Epithelial cell; Mono, monocyte; NK, natural killer cell; pDC, plasmacytoid dendritic cell; PVL, perivascular-like cell.

**
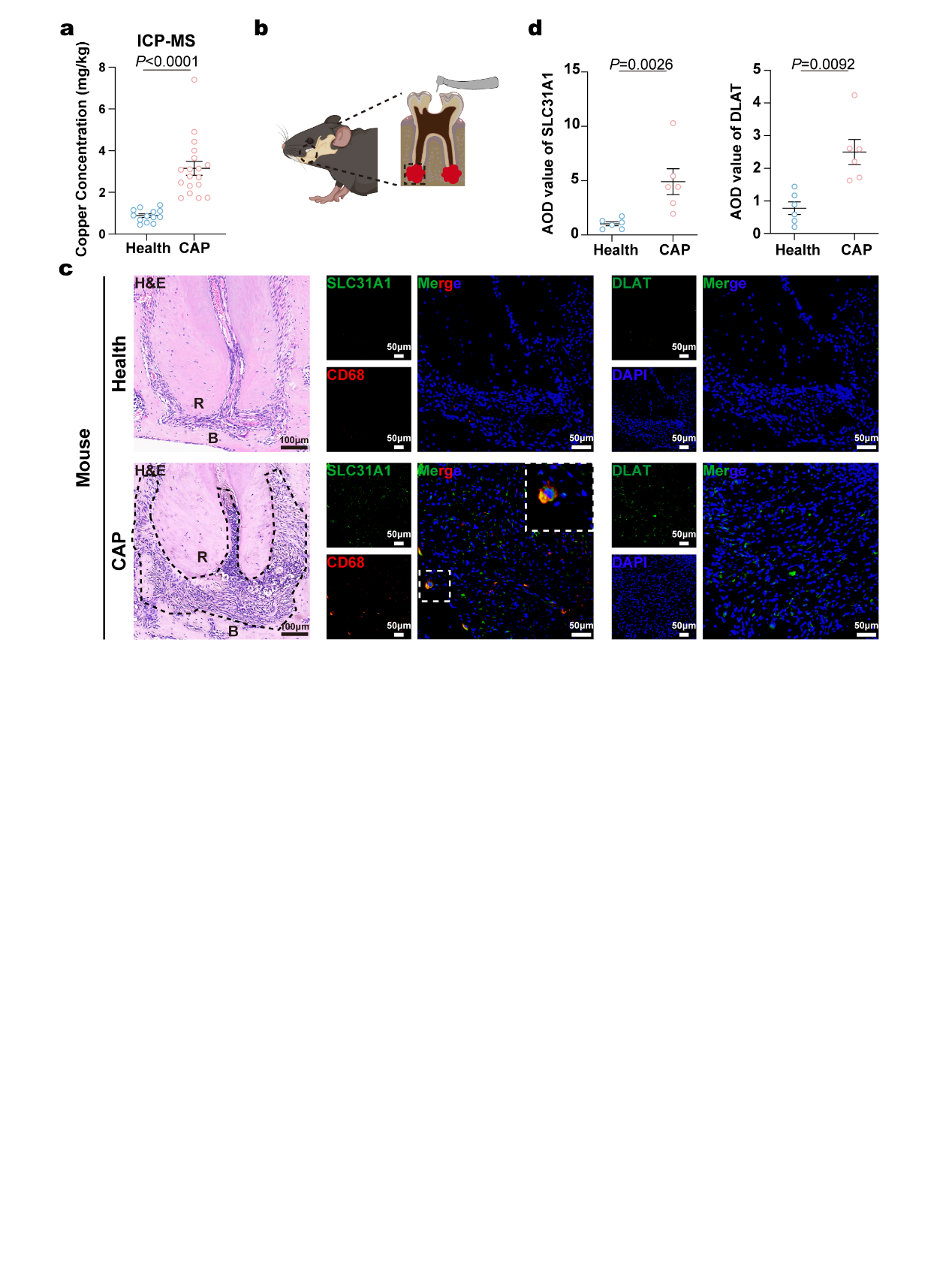
**

**Supplementary Fig 2 (a)** ICP-MS analysis of copper content in human healthy oral mucous (n=13) and CAP (n=18). **(b)** Schematic diagram of mouse model of CAP. **(c)** Consecutive slices from mouse CAP tissues and healthy apical tissues were stained with H&E, anti-SLC31A1 antibody, anti-CD68 antibody, anti-DLAT antibody and DAPI. R: root, B: bone. **(d)** Difference in the average optical density (AOD) values of SLC31A1 between mouse CAP (n=6) and healthy apical tissues (n=6). Difference in the average optical density (AOD) values of DLAT between mouse CAP (n=6) and healthy apical tissues (n=6). All error bars are mean ± SEM. *P* values were calculated by unpaired 2-tailed Student’s *t* test.

**
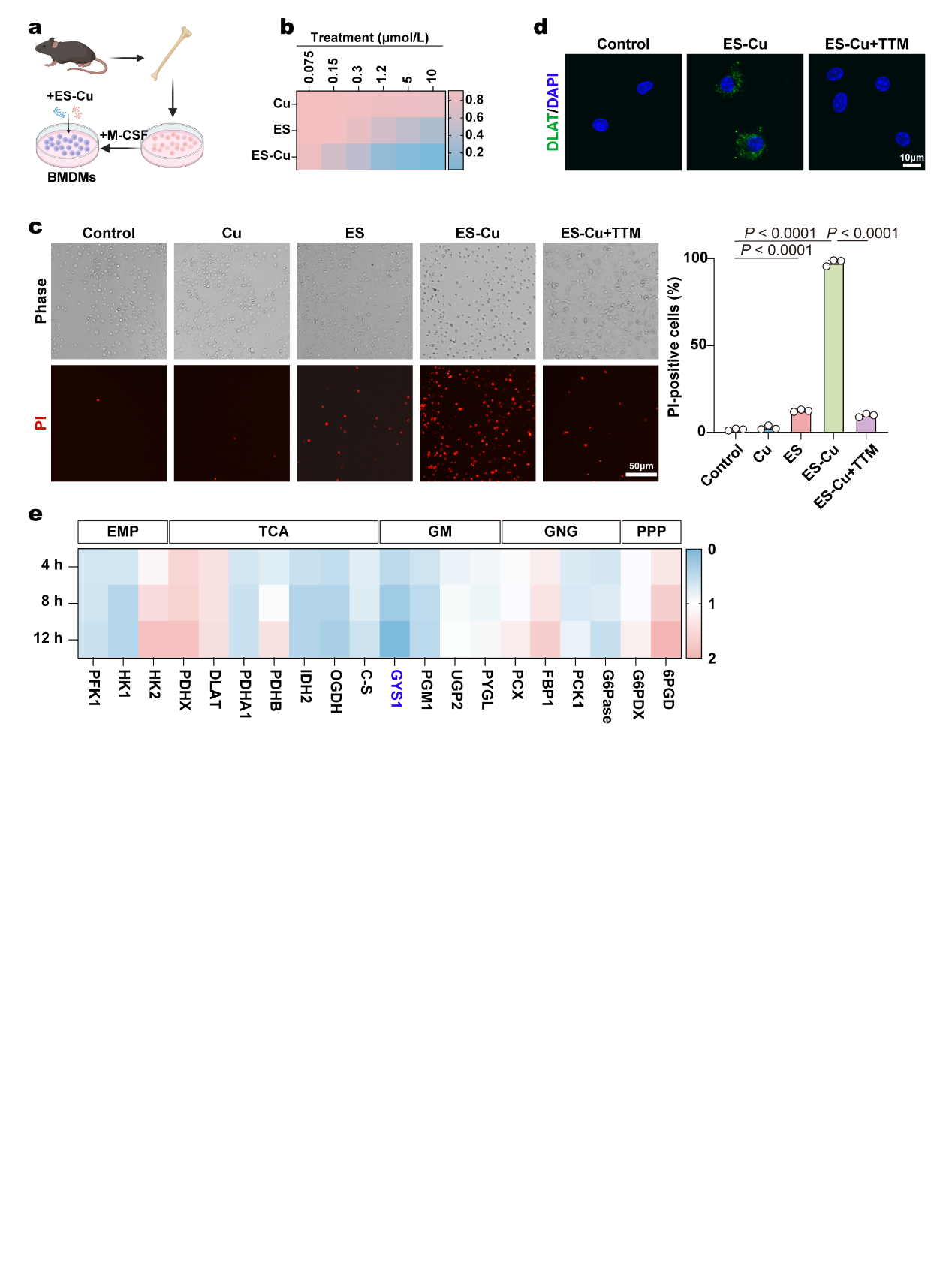
**

**Supplementary Fig 3 (a)** Schematic diagram of collecting mouse bone marrow derived macrophages (BMDMs). **(b)** Dose-dependent toxicity of copper-induced cell death of macrophages supplemented with Cu, ES and ES-Cu. Cell viability was assessed 24 h after using CCK8. **(c)** Macrophages were treated with 200 nmol/L ES, Cu for 24 h after 20 μmol/L TTM pretreatment for 30 min and then stained with propidium iodide (PI) for cell death detection. Representative images of PI-positive cells were captured and quantified. Scale bar: 50 μm. **(d)** Immunoﬂuorescence staining of DLAT in macrophages treated with ES-Cu or TTM for 12 h. Scale bar: 10 μm. **(e)** Heatmap of the expression of glucose-related enzymes in ES-Cu treated macrophages by RT-PCR. Data are from 3 independent experiments. All error bars are mean ± SEM. *P* values were calculated by 1-way ANOVA followed by Tukey’s multiple-comparison test.

**
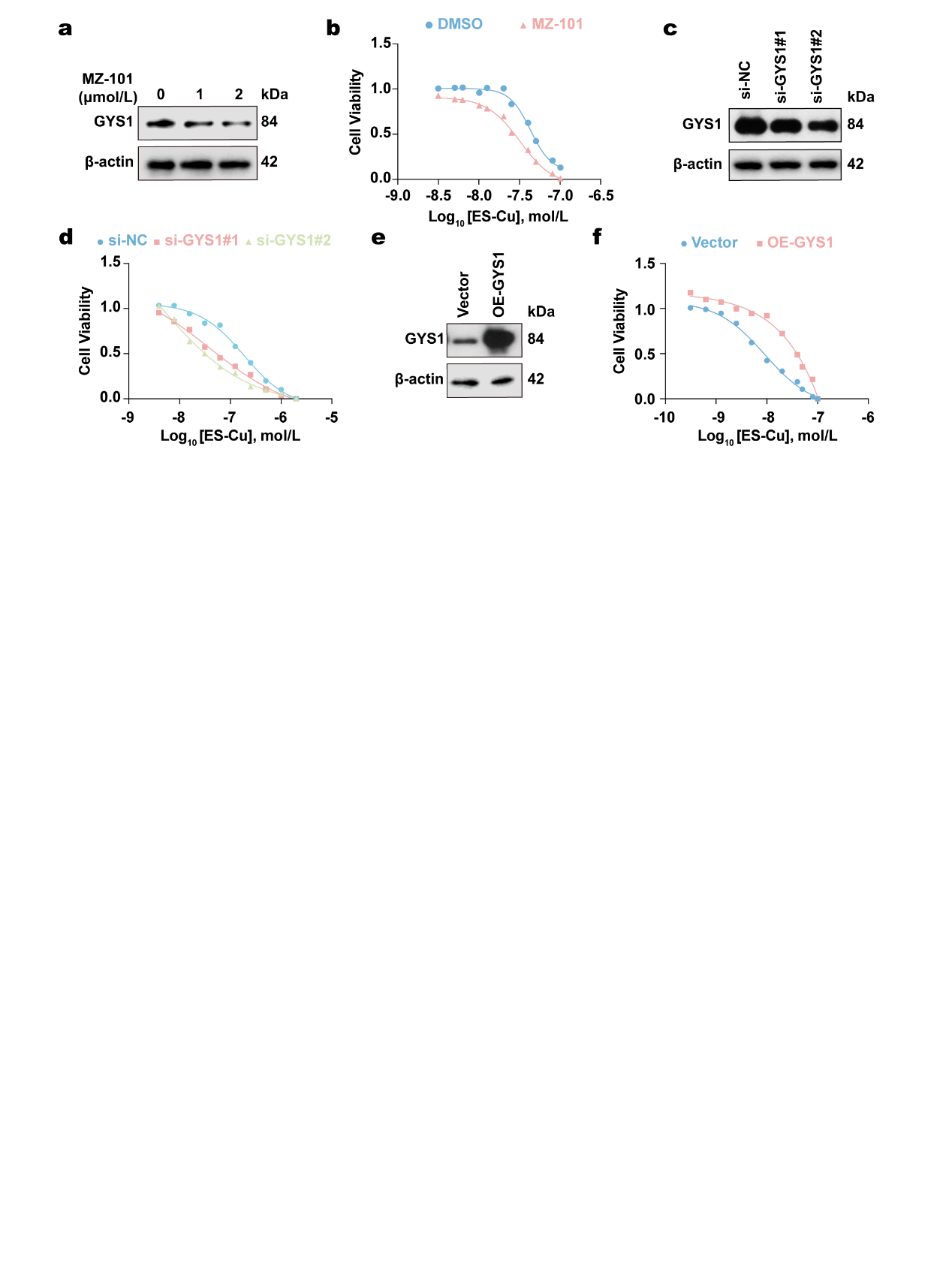
**

**Supplementary Fig 4 (a)** Macrophages were treated with ES-Cu (150 nmol/L, 1:1) for 12 h after 1 μmol/L MZ-101 pre-treatment for 30 min. Western blot showed the decrease of GYS1. **(b)** Cell viability was analyzed. **(c-d)** Macrophages were pretreated with GYS1 siRNA and then treated with ES-Cu (150 nmol/L, 1:1) for 12 h. The GYS1 level was detected by western blot (c) and the cell viability (d) was analyzed. (**e**-**f)** Macrophages were treated with ES-Cu (150 nmol/L, 1:1) after overexpressing GYS1 (e). The cell viability was analyzed (f).

**
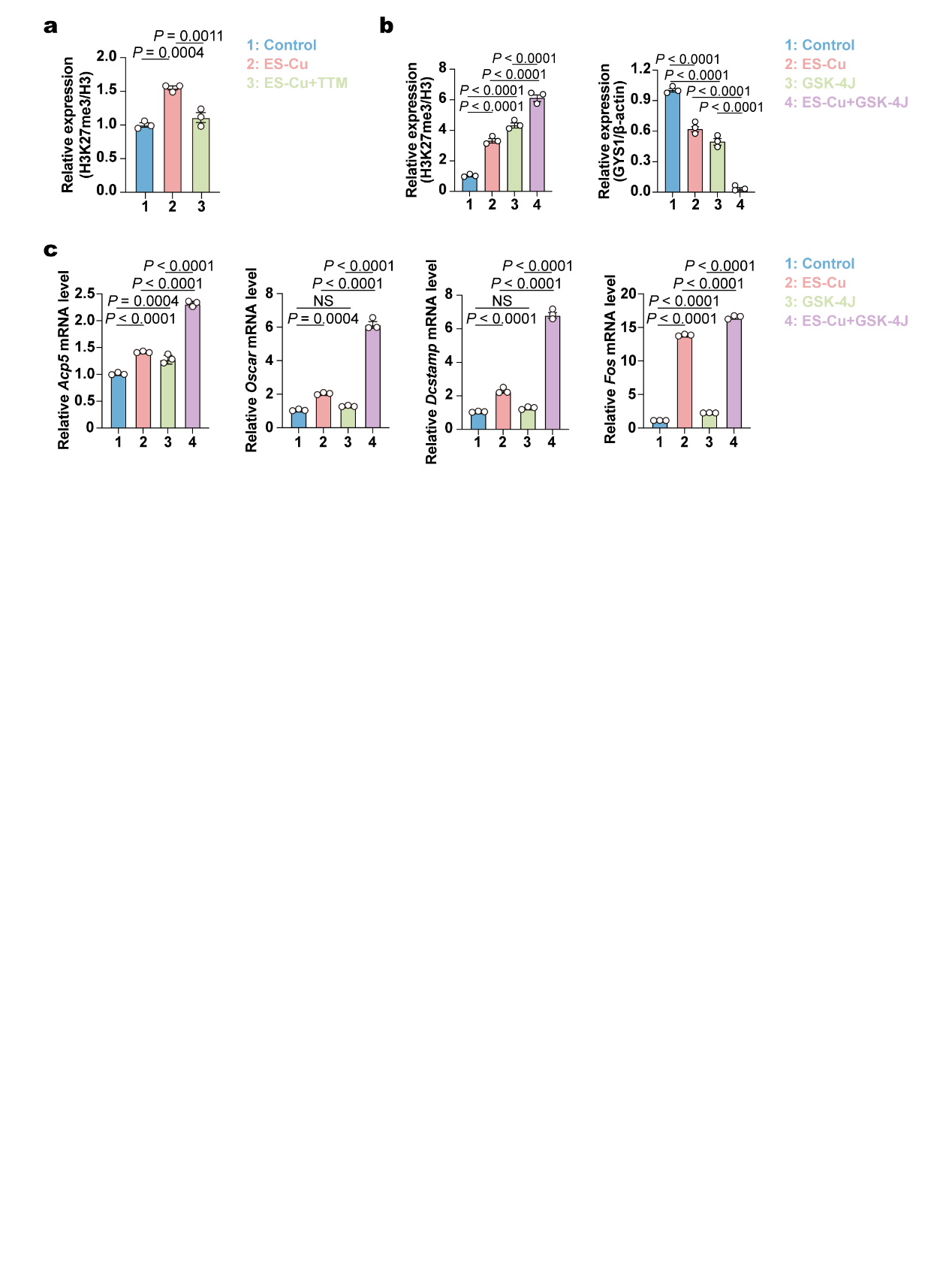
**

**Supplementary Fig 5** (**a-b**) Quantification of western blot analysis. **(c)** The mRNA levels of *Acp5*, *Oscar*, *Dcstamp* and *Fos* in macrophages treated with ES-Cu after GSK-J4 pretreatment. Data are from 3 independent experiments. All error bars are mean ± SEM. *P* values were calculated by 1-way ANOVA followed by Tukey’s multiple-comparison test.

**
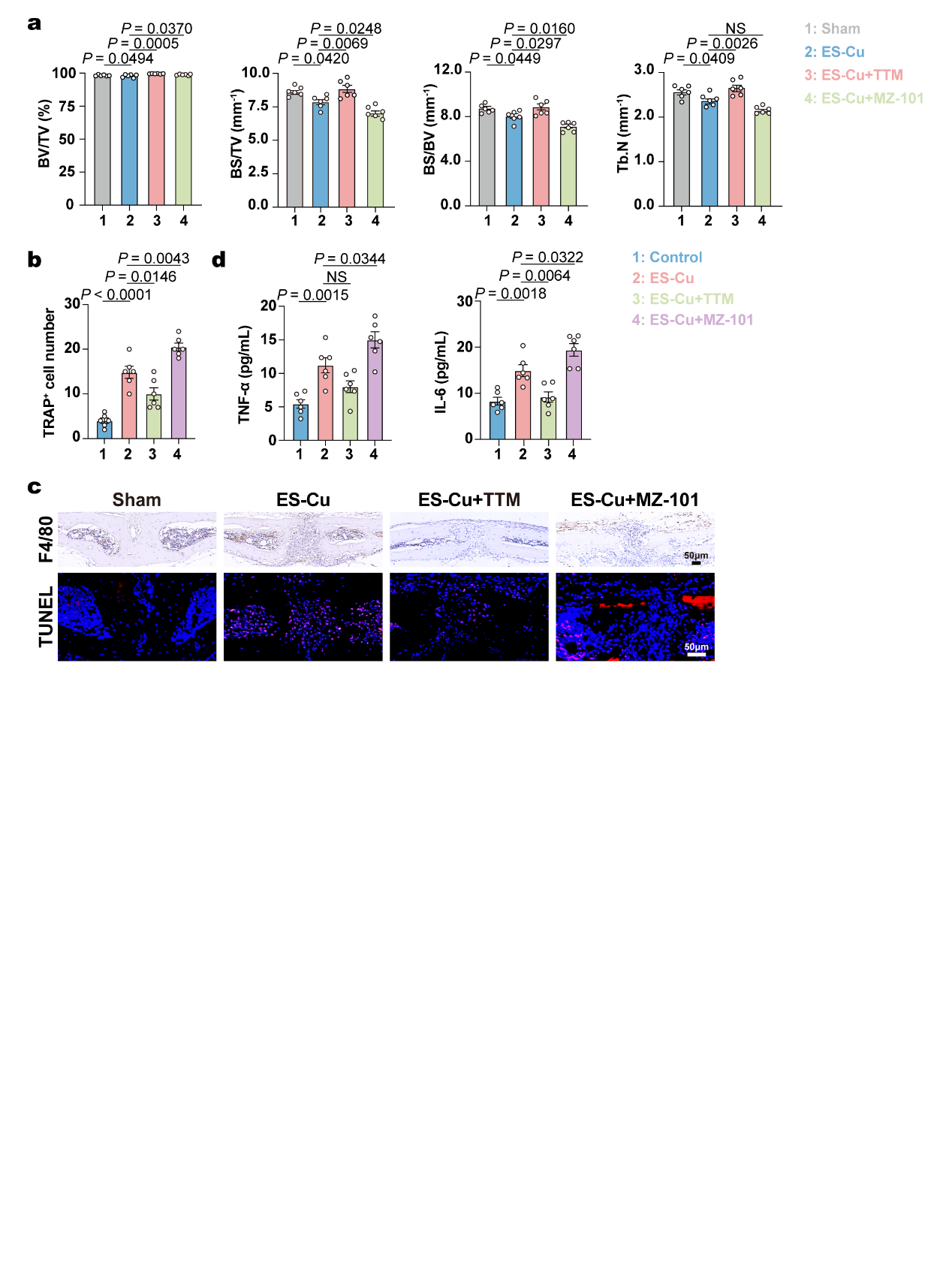
**

**Supplementary Fig 6 (a)** Micro-computed tomography (micro-CT) analysis of mouse calvarial bones. Quantification of trabecular bone volume/tissue volume (BV/TV), bone surface/ tissue volume (BS/TV), bone surface/ bone volume (BS/BV) and trabecular number (Tb. N) (n = 6). (**b**) The quantification of TRAP^+^ osteoclasts of mouse calvarial bones (n=6). **(c)** Representative images of anti-F4/80 body staining and TUNEL staining at calvarial bones. Scale bars: 50 μm (n = 6). **(d)** The levels of inflammatory cytokines TNF-α and IL-6 in mouse serum were measured by ELISA after the TTM or MZ-101 treatment in ES-Cu calvarial osteolysis models (n=6). All error bars are mean ± SEM. *P* values were calculated by 1-way ANOVA followed by Tukey’s multiple-comparison test.


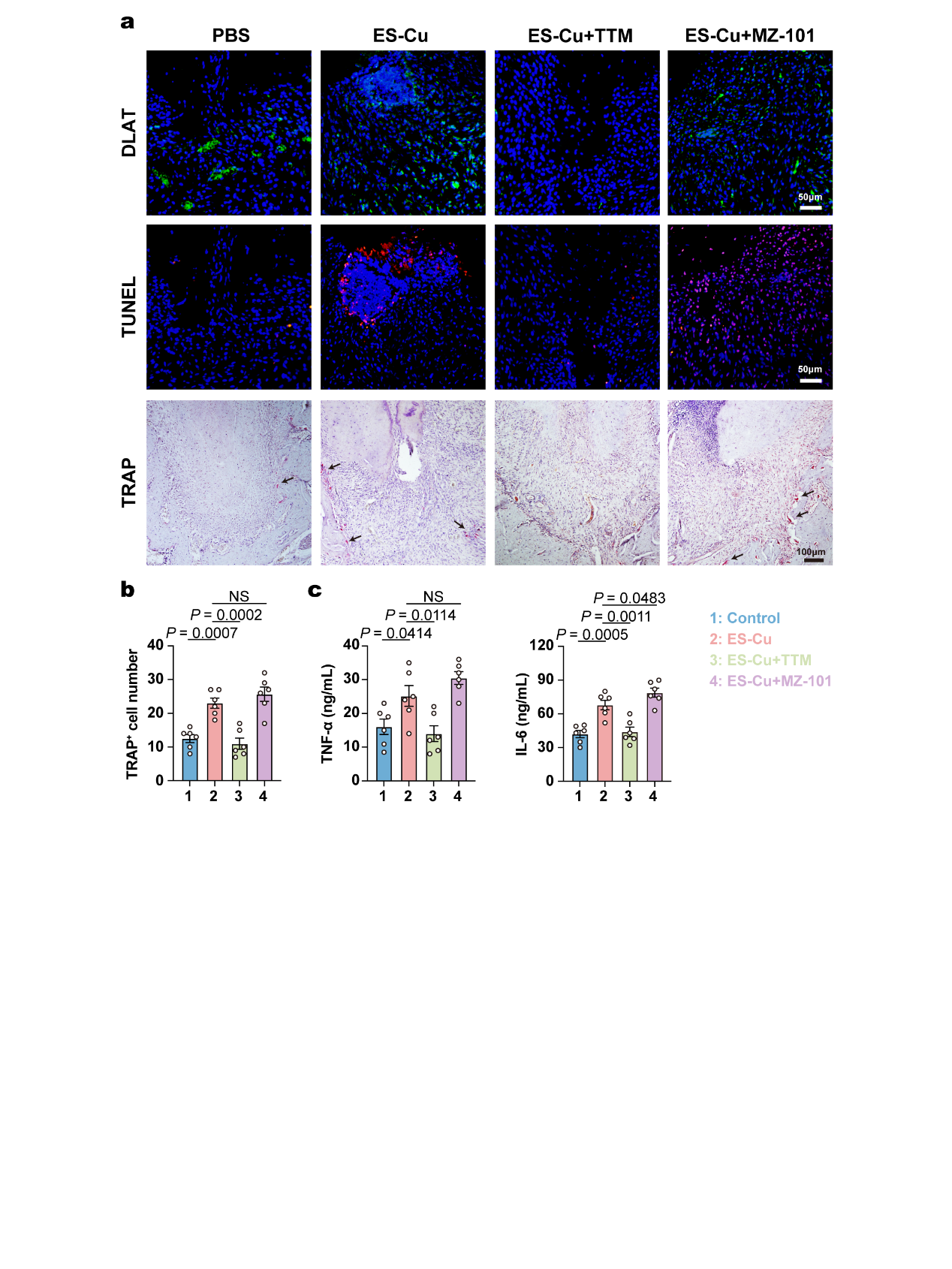


**Supplementary Fig 7 (a)** Representative images of anti-DLAT body, TUNEL staining and TRAP staining after the MZ-101 treatment in ES-Cu induced apical periodontitis rat model (n=6). **(b)** The quantification of TRAP^+^ osteoclasts in ES-Cu induced apical periodontitis rat model (n=6). **(c)** The levels of inflammatory cytokines TNF-α and IL-6 in rat serum were measured by ELISA after the MZ-101 treatment in ES-Cu induced apical periodontitis rat model (n=6). All error bars are mean ± SEM. *P* values were calculated by 1-way ANOVA followed by Tukey’s multiple-comparison test.

**Supplementary Table 1. Characteristics details of the donors for H&E and IF staining.**

| **Patients No.** | **Gender** | **Age (Years)** | **Diagnosis** |
| --- | --- | --- | --- |
| Patient 1 | Female | 31 | Health |
| Patient 2 | Female | 30 | CAP |
| Patient 3 | Male | 19 | Health |
| Patient 4 | Male | 26 | Health |
| Patient 5 | Female | 18 | Health |
| Patient 6 | Female | 48 | CAP |
| Patient 7 | Female | 31 | CAP |
| Patient 8 | Female | 35 | CAP |
| Patient 9 | Male | 28 | CAP |
| Patient 10 | Male | 20 | Health |
| Patient 11 | Male | 29 | Health |
| Patient 12 | Female | 40 | CAP |
| Patient 13 | Female | 43 | CAP |
| Patient 14 | Male | 44 | CAP |
| Patient 15 | Male | 22 | CAP |
| Patient 16 | Female | 31 | CAP |
| Patient 17 | Male | 18 | Health |
| Patient 18 | Female | 16 | CAP |
| Patient 19 | Female | 23 | Health |
| Patient 20 | Male | 54 | CAP |
| Patient 21 | Male | 47 | CAP |
| Patient 22 | Female | 52 | CAP |
| Patient 23 | Female | 34 | CAP |
| Patient 24 | Male | 32 | CAP |
| Patient 25 | Female | 26 | CAP |
| Patient 26 | Female | 28 | Health |
| Patient 27 | Female | 32 | CAP |
| Patient 28 | Male | 34 | CAP |
| Patient 29 | Male | 20 | Health |
| Patient 30 | Female | 21 | Health |
| Patient 31 | Male | 32 | Health |
| Patient 32 | Female | 28 | Health |

**Supplementary Table 2. Primers for qPCR.**

| Gene | Primer | Primer sequence 5’-3’ |
| --- | --- | --- |
| *Mouse Pfk1* | Forward | TGTGGTCCGAGTTGGTATCTT |
|  | Reverse | GCACTTCCAATCACTGTGCC |
| *Mouse Hk1* | Forward | CGGAATGGGGAGCCTTTGG |
|  | Reverse | GCCTTCCTTATCCGTTTCAATGG |
| *Mouse Hk2* | Forward | ATGCGTAATGTGGAACTGGTG |
|  | Reverse | GCTGATCATCTTCTCAAACCTCTG |
| *Mouse Pdhx* | Forward | GTGGTCACCTTAGATGCAAACG |
|  | Reverse | CCTGTTTCCAATCTTCCCCTTC |
| *Mouse Dlat* | Forward | GACCAGCTTAAAGCCACAGG |
|  | Reverse | AGAGGACTAACGAACACCCTT |
| *Mouse Pdha1* | Forward | GAAATGTGACCTTCATCGGCT |
|  | Reverse | TGATCCGCCTTTAGCTCCATC |
| *Mouse Pdhb* | Forward | AGGAGGGAATTGAATGTGAGGT |
|  | Reverse | ACTGGCTTCTATGGCTTCGAT |
| *Mouse Idh2* | Forward | GGAGAAGCCGGTAGTGGAGAT |
|  | Reverse | GGTCTGGTCACGGTTTGGAA |
| *Mouse Ogdh* | Forward | AGGGCATATCAGATACGAGGG |
|  | Reverse | CTGTGGATGAGATAATGTCAGCG |
| *Mouse C-s* | Forward | GGACAATTTTCCAACCAATCTGC |
|  | Reverse | TCGGTTCATTCCCTCTGCATA |
| *Mouse Gys1* | Forward | CACAGAACGGTTGTCGGACTTG |
|  | Reverse | AGGTGAAGTGGTCTGGAAAGGC |
| *Mouse Pgm1* | Forward | AGCCAATGACCCAGATGCTGAC |
|  | Reverse | TCCAGGAAGTGAAGAGCCACCA |
| *Mouse Ugp2* | Forward | CTGATGAACCCACCCAATGGGA |
|  | Reverse | GAGCGATTTCCACCAGTCTCAG |
| *Mouse Pcx* | Forward | CTGAAGTTCCAAACAGTTCGAGG |
|  | Reverse | CGCACGAAACACTCGGATG |
| *Mouse Fbp1* | Forward | TGCTGAAGTCGTCCTACGCTAC |
|  | Reverse | TTCCGATGGACACAAGGCAGTC |
| *Mouse Pck1* | Forward | GGCGATGACATTGCCTGGATGA |
|  | Reverse | TGTCTTCACTGAGGTGCCAGGA |
| *Mouse Pygl* | Forward | GGCAGAAGTGGTGAACAATGACC |
|  | Reverse | TCCGATAGGTCTGTGGCTGGAA |
| *Mouse G6pase* | Forward | AGGTCGTGGCTGGAGTCTTGTC |
|  | Reverse | GTAGCAGGTAGAATCCAAGCGC |
| *Mouse G6pdx* | Forward | GACCAAGAAGCCTGGCATGTTC |
|  | Reverse | AGACATCCAGGATGAGGCGTTC |
| *Mouse 6Pdg* | Forward | CATCGCTGCAAAAGTGGGAACC |
|  | Reverse | AGCCTCACAGATGAGCTGCATG |
| *Mouse Acp5* | Forward | CACTCCCACCCTGAGATTTGT |
|  | Reverse | CCCCAGAGACATGATGAAGTCA |
| *Mouse Oscar* | Forward | CCTAGCCTCATACCCCCAG |
|  | Reverse | CGTTGATCCCAGGAGTCACAA |
| *Mouse Dcstamp* | Forward | GGGGACTTATGTGTTTCCACG |
|  | Reverse | ACAAAGCAACAGACTCCCAAAT |
| *Mouse Fos* | Forward | CGGGTTTCAACGCCGACTA |
|  | Reverse | TTGGCACTAGAGACGGACAGA |
| *Mouse Actb* | Forward | GGCTGTATTCCCCTCCATCG |
|  | Reverse | CCAGTTGGTAACAATGCCATGT |

**Supplementary 3. Primers for ChIP.**

| Gene | Primer | Primer sequence 5’-3’ |
| --- | --- | --- |
| ChIP-GYS1 primer1 | Forward | GCTGTGGGAGCCTTCACCTTCCTCT |
|  | Reverse | GGCATATTGGGCCTGGCACTAGCTC |
| ChIP-GYS1 primer2 | Forward | CTTCCTCCACATCCAGGAGTCCAGG |
|  | Reverse | GTCGCCAAGGAGGGCTTGGGAGGAC |
| ChIP-GYS1 primer3 | Forward | GTCCTCCCAAGCCCTCCTTGGCGAC |
|  | Reverse | TTCTTAGAGAGTCTGAAGGAGGGAG |
